# The circadian clock gene Bmal1 facilitates cisplatin-induced renal injury and hepatization

**DOI:** 10.1038/s41419-020-2655-1

**Published:** 2020-06-10

**Authors:** Min Zha, Ting Tian, Weilong Xu, Su Liu, Jia Jia, Lijuan Wang, Qianhua Yan, Nan Li, Jiangyi Yu, Liji Huang

**Affiliations:** Affiliated Hospital of Nanjing University of Chinese Medicine, Jiangsu Province Hospital of Chinese Medicine, Nanjing, Jiangsu China

**Keywords:** Nephrons, Toxin-induced nephropathy

## Abstract

Cisplatin is one of the most potent chemotherapy drugs to treat cancers, but its clinical application remains limited due to severe nephrotoxicity. Several approaches have been developed to minimize such side effects, notably including chronotherapy, a well-known strategy based on the circadian clock. However, the component of the circadian clock machinery that particularly responses to the cisplatin stimulation remains unknown, including its functions in cisplatin-induced renal injury. In our present study, we demonstrated that Bmal1, as a key clock gene, was induced by the cisplatin stimulation in the mouse kidney and cultured human HK-2 renal cells. Gain- and loss-of-function studies indicated that Bmal1 facilitated cisplatin-induced renal injury both in vivo and in vitro, by aggravating the cell apoptotic process. More importantly, RNA-seq analysis revealed that Bmal1 triggered the expression of hallmark genes involved in renal hepatization, a critical event accompanied by the injury. At the molecular level, Bmal1 activated the transcription of hepatization-associated genes through direct recruitment to the E-box motifs of their promoters. Our findings suggest that Bmal1, a pivotal mediator induced renal injury in response to cisplatin treatment, and the therapeutic intervention targeting Bmal1 in the kidney may be a promising strategy to minimize the toxic side-effects of cisplatin in its clinical applications.

## Introduction

Cisplatin, a platinum-based chemotherapeutic agent, has been widely used in the treatment of various solid tumors, including lung, breast, esophageal, ovarian, and pancreatic cancers^1–5^. Despite the efficiency of cisplatin, the undesirable side effects occurring in multiple tissues, especially in the kidney, limit its clinical application^6^. Specifically, cisplatin is incorporated into renal cells by organic cation transporter 2 (OCT2), and then forms DNA adducts, resulting in the activation of apoptotic signals and the renal cell death^7,8^. Even though the toxicity of cisplatin remains well recognized, the substitutes of cisplatin fail to satisfactorily meet the clinic demands. Until now, only two (carboplatin and oxaliplatin) have been approved worldwide and a few analogs have entered in clinical trials^9^. Among which, the carboplatin is a second generation platinum agent that can be used for cancer patients with impaired kidney function, while oxaliplatin is known to be functional in cisplatin‐resistant cancer^10^. Unfortunately, these drugs also cause side-effects, including myelosuppression and neurological disorders^11^, hence consolidating the position of cisplatin as the first-line drug in the therapeutic application. Resolving such a paradox is necessary to explore novel strategies to minimize cisplatin-induced toxicity, including renal injury while keeping its pharmacological functions intact.

Currently, several approaches have been developed to minimize the toxicity of cisplatin, notably including chronotherapy and hydration regimens/supplementation^12,13^. Chronotherapy is defined by the administration of therapeutic agents at the right time, according to the biological rhythms of the host, and has been proven to functionally reduce the renal injury induced by cisplatin both in humans and in model animals^12,14–16^. For example, cisplatin-induced nephrotoxicity, as well as related complications, including bleeding, infection, and transfusions attenuate in patients treated with cisplatin in the afternoon than at other times^14^. Therefore, the administration time window is critical for the toxicity of cisplatin and should be carefully determined to alleviate the undesirable side effects of cisplatin.

As mentioned above, the concept of chronotherapy origins from the virtue of the circadian clocks, which entrain physiological processes in a 24-h periodicity. A general transcriptional/translational circuit regulates the circadian system in mammals^17^. In the core loop, two basic helix-loop, helix PAS transcriptional factors, brain and muscle ARNT-like 1 (Bmal1), and circadian locomotor output cycles kaput (Clock), form the heterodimers to activate the expression of circadian repressors, including Period (Per) 1/2/3 and Cryptochrome (Cry) 1/2, via directly binding to the E-box motif^18^. Per and Cry proteins, in turn, form a dimeric complex and further translocates into the nucleus and inhibit Clock/Bmal1-mediated transcription^19,20^. Of note, 43% of protein-coding genes in the mouse are demonstrated to exhibit circadian rhythmicity^21–23^. Among these, over 170 of these clock-controlled genes (CCGs) serve as the drug targets. In particular, 56 of these 170 genes are targets of the top 100 best-selling drugs in the US, implicating that the circadian clock machinery is extensively involved in the chronopharmacology^21^. This evidences that the renal toxicity of cisplatin demonstrates a circadian difference and such a time-dependent discrepancy may cause the rhythmicity of CCGs (Cyp2e1, Oct2, and Mate1) to involve in cisplatin metabolism and excretion^24,25^. On the other hand, reports state that cisplatin disrupts the rhythmicity of the circadian clocks both in the liver and kidney of the mouse^26^. However, it remains largely unknown which component in the circadian clock system specifically responses to the cisplatin stimulation and how it functions in the cisplatin-induced renal injury.

In our present study, we aim to explore the potential clock gene participating in the renal toxicity of cisplatin and the possible mechanism for this process. Our results demonstrated that Bmal1, as a key clock component, responded to the cisplatin stimulation in mouse kidney and cultured human HK-2 renal cells. Gain- and loss-of-function studies indicated that Bmal1 aggravated cisplatin-induced renal tubular cell apoptosis both in vivo and in vitro. More importantly, RNA-seq analysis revealed that Bmal1 triggered the expression of hallmark genes involved in renal hepatization. Mechanistically, Bmal1 activated the transcription of hepatization-associated genes through direct recruitment to the E-box motifs of their promoters. Taken together, our findings suggested that Bmal1 is a key mediator to induce renal injury in response to cisplatin treatment and the therapeutic intervention targeting Bmal1 in the kidney may be a promising strategy to minimize the toxic side-effects of cisplatin in its clinical applications.

## Results

### Cisplatin induces renal injury in an administration time-dependent manner

**Fig. 1:**
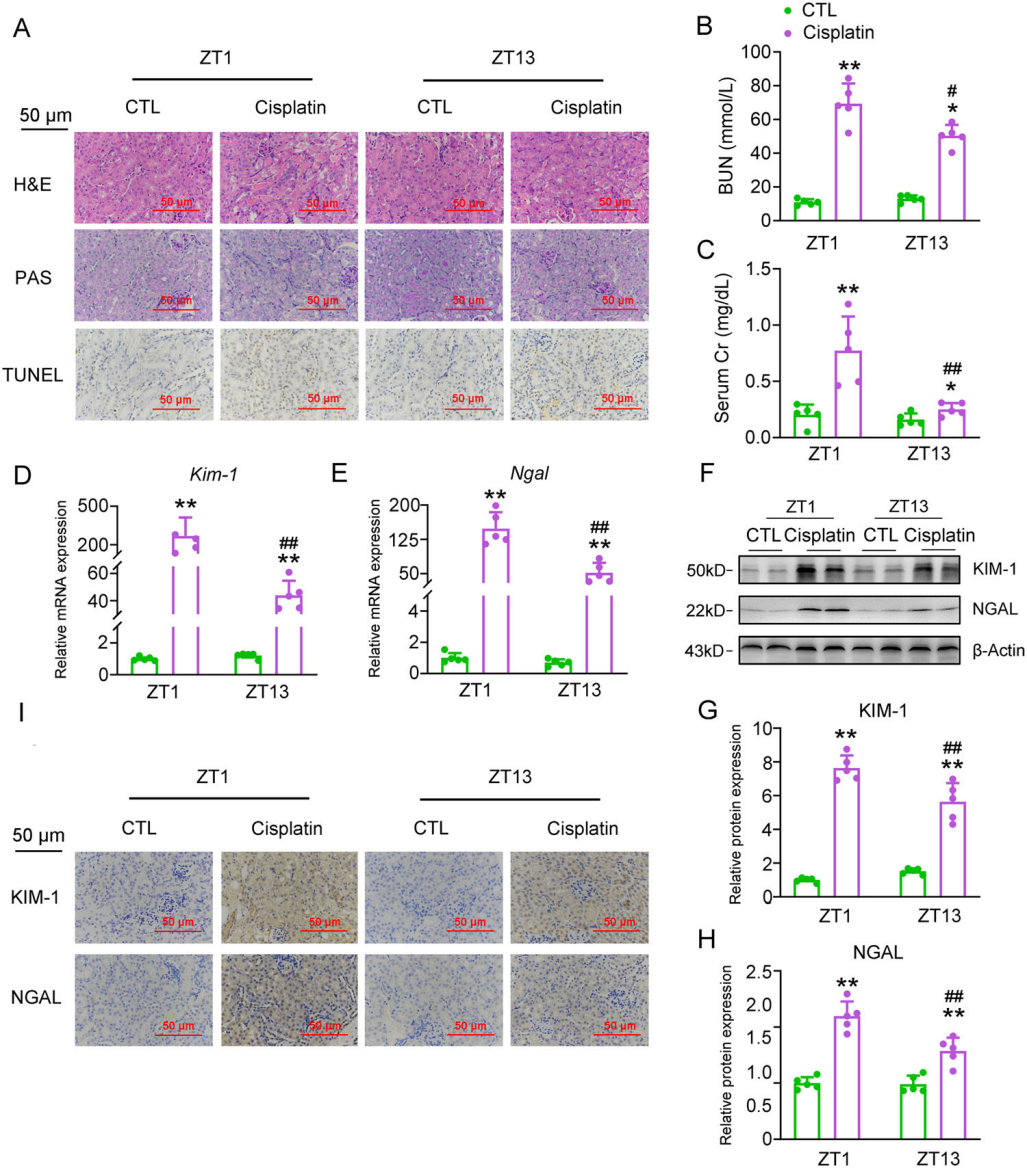
Cisplatin induces renal injury in an administration time-dependent manner. Mice were injected with cisplatin (20 mg/kg) or equivalent volume of saline at ZT1 or ZT13, respectively. In all, 72 h thereafter, mouse kidney and serum samples were collected for the following experiments. *n* = 5 for each group. **a** H&E, PAS staining and TUNEL assays of kidney sections. **b**, **c** BUN and serum Cr levels. **d**, **e** RT-qPCR analyses of renal *Kim-1* and *Ngal* mRNA expression. **f** Western blot analysis of renal Kim-1 and Ngal protein expression. **g, h** Quantitative data of panel. **f**. *P < 0.05 and **P < 0.01 vs. CTL group, ^#^*P* < 0.05 and ^##^*P* < 0.01 vs. ZT1 CTL group. **i** IHC analyses of Kim-1 and Ngal proteins. All values are presented as the mean ± SD.

Of note, the circadian toxicity of cisplatin has been recognized, and it is traditionally believed that OCT2 rhythmicity regulated by Clock and the downstream PPARα contributes importantly to the circadian toxicity of cisplatin^24^. However, such a regulation involves multiple steps and is indirect, therefore it is still unknown whether a certain clock gene directly influences cisplatin nephrotoxicity in a circadian manner. To answer this question, we first determine if cisplatin differently induces the renal injury by the administration time window. We treated mice with a single intraperitoneal (i.p.) injection of cisplatin at the dose of 20 mg/kg at the *Zeitgeber* Time (ZT) 1 and ZT13 (ZT0 is the time of lights on), respectively, which represent two typical time points of the light-dark phases switch. As shown in Fig. 1a, histological staining and immunohistochemistry (IHC) analyses revealed that cisplatin injection induced significant tubular injury in the mouse kidney, evidenced by the tubular dilatation, cast formation, brush border loss, and increased population of TUNEL-positive cells. Consistently, the serum levels of two classic markers for the renal injury, including blood urea nitrogen (BUN) and creatinine (Cr), dramatically increased after the cisplatin injection (Fig. 1b, c). At the molecular level, the expression of tubular injury-related genes, Kim-1 and Ngal, was induced both at the transcriptional and translational levels by the cisplatin treatment (Fig. 1d–i). Cisplatin when injected at different time points showed a remarkable time-dependent discrepancy. We found that injection of cisplatin at ZT1 caused more severe pathological changes in the kidney than that occurred at ZT13, evidenced by higher serum levels BUN and Cr, as well as higher expression levels of Kim-1 and Ngal in the kidney (Supplementary Table 1). The above results suggest that the renal toxicity of cisplatin manifest a diurnal variation, and the elements in the circadian clock machinery may involve in chronotoxicity.

### Cisplatin regulates renal clock gene expression both in vivo and in vitro

**Fig. 2:**
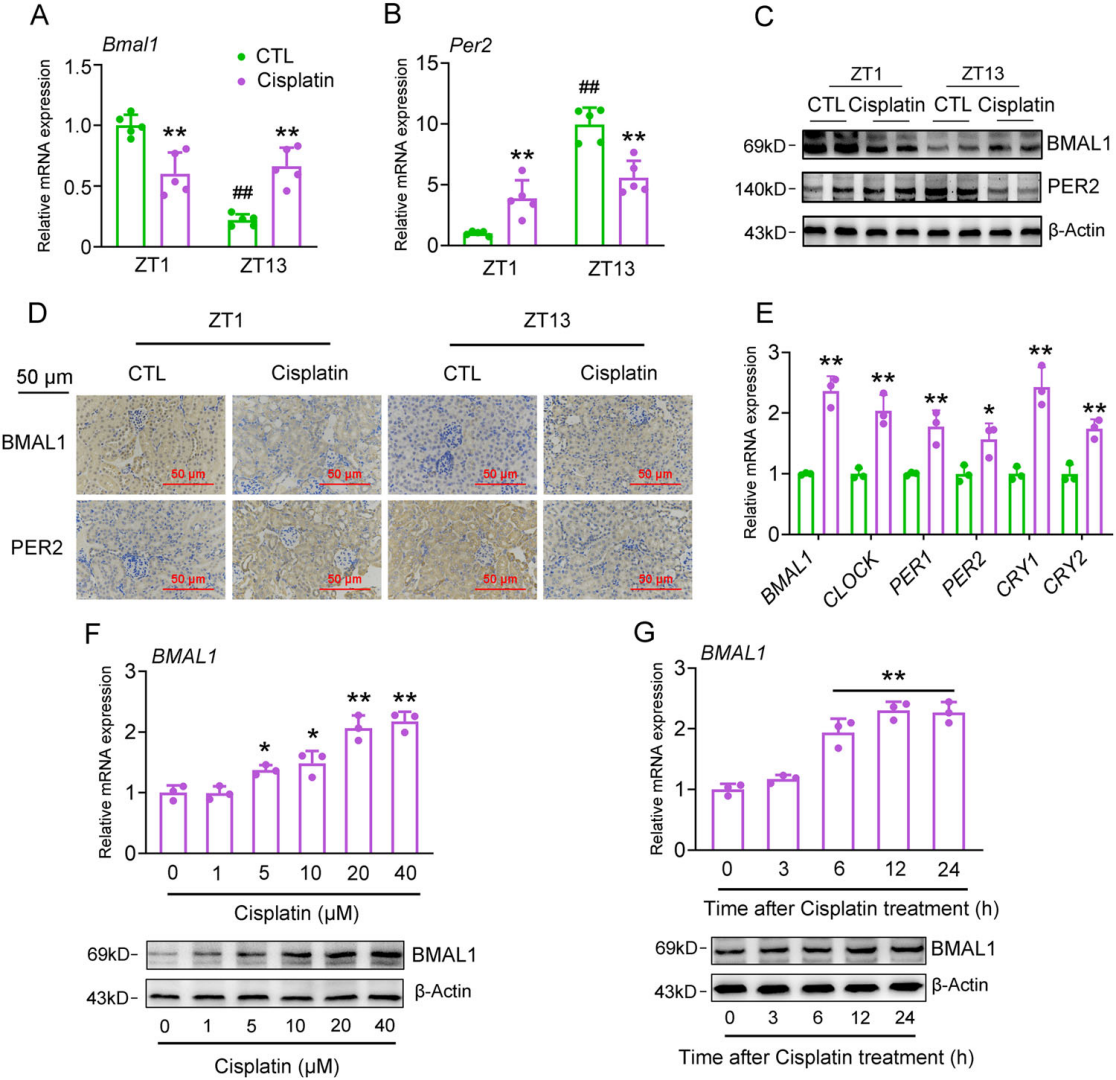
Cisplatin regulates renal clock gene expression both in vivo and in vitro. **a**, **b** RT-qPCR analyses of renal *Bmal1* and *Per2* mRNA expression. **P < 0.01 vs. CTL group, ^##^*P* < 0.01 ZT13 vs. ZT1 group. **c, d** Western blot and IHC analyses of Bmal1 and Per2 protein expression. *n* = 5 for each group. **e** RT-qPCR analyses of clock gene expression in HK-2 cells treated with either 20 μM cisplatin or vehicle for 24 h. *n* = 3, *P < 0.05 and **P < 0^.^01 vs. CTL group. **f** RT-qPCR and western blot analyses of BMAL1 expression in HK-2 cells treated with cisplatin at indicated doses for 24 h. *n* = 3, *P < 0.05 and **P < 0.01 vs. Vehicle group. **g** RT-qPCR and western blot analyses of BMAL1 expression in HK-2 cells treated with 20 μM cisplatin for indicated times. *n* = 3, **P < 0.01 vs. 0 h. All values are presented as the mean ± SD.

To confirm the participation of clock genes in mediating the chronotoxicity of cisplatin, we next assessed the mRNA expression levels of core clock genes in the kidney of cisplatin-treated mice. Our results indicated that cisplatin injection impaired the normal fluctuation of their expression at ZT1 and ZT13 (Fig. 2a–d, Fig. S1A–D). We also evaluated the effects of cisplatin on clock gene expression in cultured HK-2 cells and found that 24-h cisplatin treatment increased the mRNA expression levels of all examined clock genes (Fig. 2e). More importantly, Bmal1 is most responsive to the cisplatin treatment among all these clock genes, we found that Bmal1 expression was reduced in response to the injection of cisplatin at ZT1, while was induced corresponding to the ZT13 injection in vivo, leading to a bidirectional variation. In addition, cisplatin significantly induces Bmal1 expression in vitro. In the following experiments, we further specified the regulation of Bmal1 expression by cisplatin. Cisplatin increased the mRNA and protein expression levels of *BMAL1* based on dose and time in HK-2 cells (Fig. 2f, g).

### Overexpression of Bmal1 accelerates cisplatin-induced renal injury

**Fig. 3:**
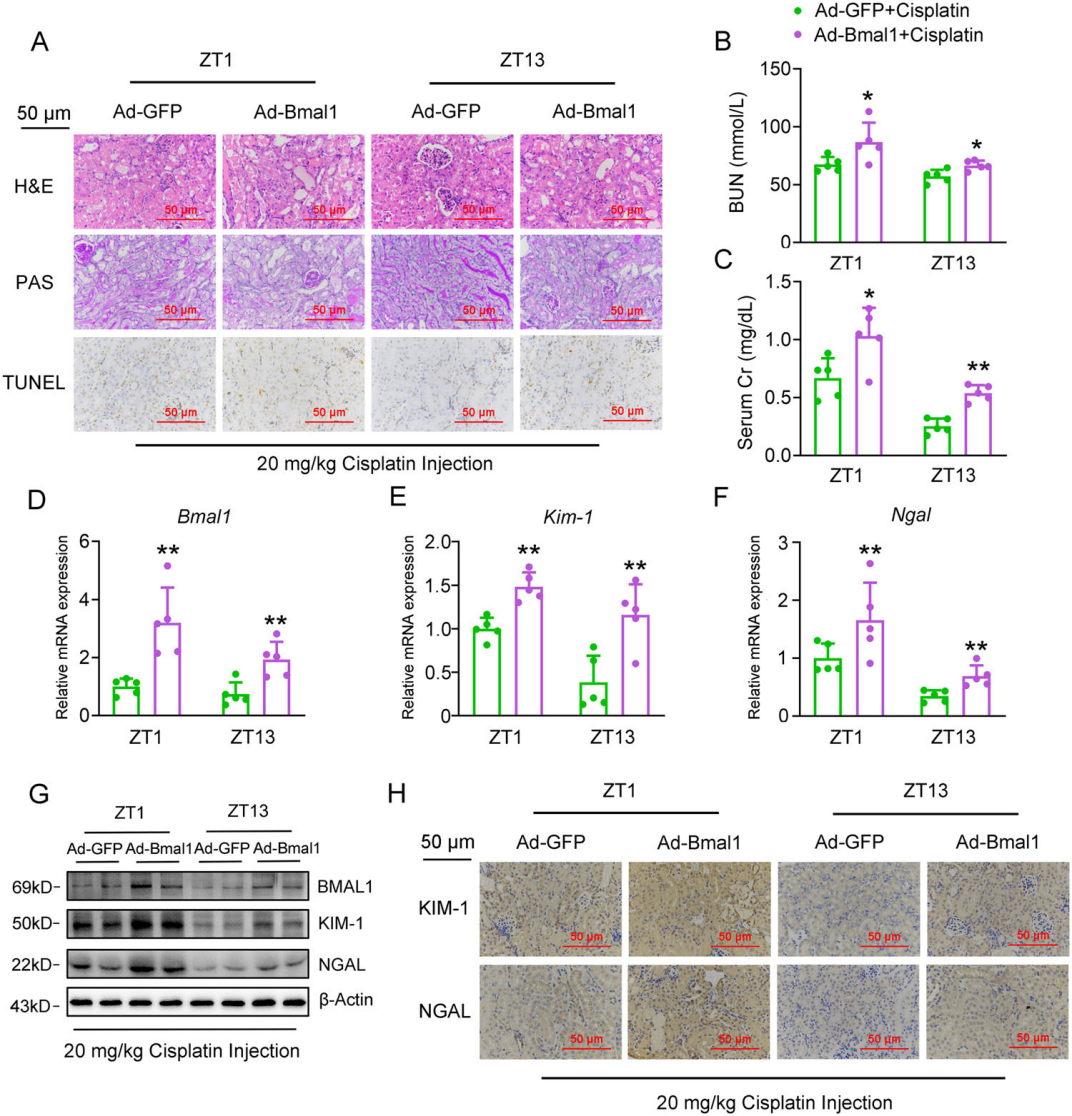
Overexpression of Bmal1 accelerates cisplatin-induced renal injury in vivo. Mice were transduced with adenovirus expressing either Bmal1 CDS domain or GFP via tail-vein injection for 4 days, followed by cisplatin treatment at ZT1 or ZT13 for another 72 h. Mouse kidney and serum samples were collected for the following experiments. *n* = 5 for each group. **a** H&E, PAS staining and TUNEL assays of kidney sections. **b**, **c** BUN and serum Cr levels. **d**–**g** RT-qPCR and western blot analyses of renal expression levels of Bmal1, Kim-1 and Ngal. **h** IHC analyses of Kim-1 and Ngal protein expression from kidney sections. *P < 0.05 and **P < 0.01 vs. Ad-GFP + cisplatin group. All values are presented as the mean ± SD.

To examine the pathophysiological role of Bmal1 in cisplatin-induced renal injury in vivo, we constructed mice with nephron-specific Bmal1 overexpression by using an adenovirus system carrying a Bmal1 CDS domain. As shown in Fig. 3a, all the pathological characteristics, including tubular dilatation, cast formation, brush border loss, and the TUNEL-positive cells, significantly aggravated in the kidney of cisplatin-treated mice with Bmal1 overexpression. Meanwhile, these mice had higher serum levels of BUN and Cr than with mice treated by cisplatin (Fig. 3b, c), and the expression levels of Kim-1 and Ngal correspondingly increased in the kidney in response to Bmal1 exogenous expression (Fig. 3d–h, Fig. S3a–c). Supplementary Table 2 shows the statistical comparison. Following the in vivo results, we found that Bmal1 overexpression increased the cisplatin-induced apoptosis of HK-2 cells by 2.9% (Figs. S2A and S3D) and confirmed these observations by the TUNEL assays (Figs. S2B and S3E). As the apoptotic markers, the expression ratio between BAX and BCL2, as well as the expression levels of cleaved-Caspase3, was increased in parallel (Figs. S2C, D and S3F–H). We also found that Bmal1 overexpression further upregulated the expression levels of KIM-1 and NGAL in cisplatin-treated HK-2 cells (Figs. S2C, D and S3I, J). In addition, to specify the regulatory role of Bmal1 in the apoptosis of HK-2 cells, we overexpressed another clock component Per2 in these cells and found that Per2 modestly affected the HK-2 apoptosis as evidenced by TUNEL assays (Fig. S4A, B, *P* = 0.4910). This result was further confirmed by unaltered mRNA expression levels of cell apoptotis- and tubular injury-related genes (Fig. S4C–F, *P* = 0.2889 for *BAX/BCL2* ratio, *P* = 0.1645, for *KIM-1*, *P* = 0.1966 for *NGAL*)

Collectively, these findings provide strong evidence to show that Bmal1 is a bona fide culprit in cisplatin-induced tubular injury.

### Knockdown of Bmal1 alleviates cisplatin-induced renal injury

**Fig. 4:**
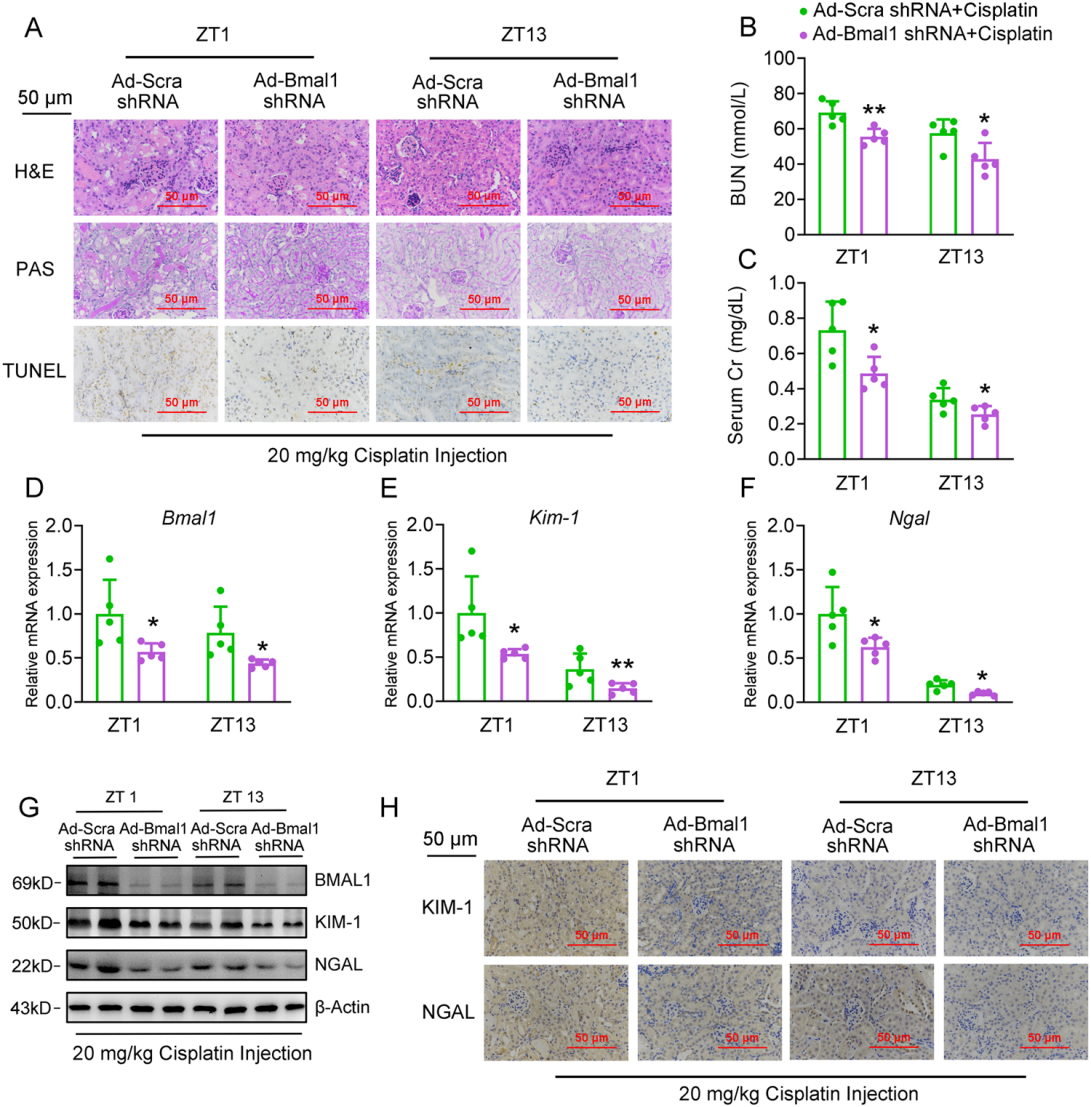
Knockdown of Bmal1 alleviates cisplatin-induced renal injury in vivo. Mice were transduced with adenovirus expressing either Bmal1 shRNA or scramble shRNA via tail-vein injection for 4 days, followed by cisplatin treatment at ZT1 or ZT13 for another 72 h. Mouse kidney and serum samples were collected for the following experiments. *n* = 5 for each group. **a** H&E, PAS staining and TUNEL assays of kidney sections. **b**, **c** BUN and serum Cr levels. **d**–**g** RT-qPCR and western blot analyses of renal expression levels of Bmal1, Kim-1 and Ngal. **h** IHC analyses of Kim-1 and Ngal from kidney sections. *P < 0.05 and **P < 0.01 vs. Ad-Scramble shRNA+ cisplatin group. All values are presented as the mean ± SD.

We next adopted a loss-of-function strategy to confirm the facilitation of Bmal1 in the cisplatin-induced renal injury. The nephron-specific knockdown of Bmal1 ameliorated cisplatin-induced tubular injury with significant attenuation of tubular dilatation, cast formation, brush border loss, and less TUNEL-positive cells (Fig. 4a). Consistently, the serological analysis revealed that cisplatin-induced increase of BUN and serum Cr levels decreased in mice with nephron-specific Bmal1 knockdown (Fig. 4b, c). At the molecular level, knockdown of Bmal1 retarded the cisplatin-induced upregulation of KIM-1 and NGAL expression (Fig. 4d–h, Fig. S6A–C). Interestingly, we noticed that the severity of renal injury positively correlated to the expression levels of Bmal1. In Figs. 1 and 2, we already found that the expression of Bmal1 at ZT13 lower than that at ZT1, and injection of cisplatin at ZT 13 led to a milder kidney injury. Here, we indicated that if Bmal1 expression further decreases the shRNA oligonucleotides at ZT13, and the pathological changes induced by cisplatin treatment completely diminished when compared to the control mice (Supplementary Table 3). For in vitro analyses, shRNA-mediated knockdown of Bmal1 decreased the cisplatin-induced cell apoptosis (Figs. S5A, B and S6D, E), when assessed by the Flow cytometry and TUNEL assays. In agreement with these data, knockdown of Bmal1 markedly decreased the expression ratio between BAX and BCL2, repressed cleaved-Caspase3, and reduced the expression levels of Kim-1 and Ngal (Figs. S5C, D and S6F–J).

### Bmal1 is a key activator for cisplatin-induced renal hepatization

**Fig. 5:**
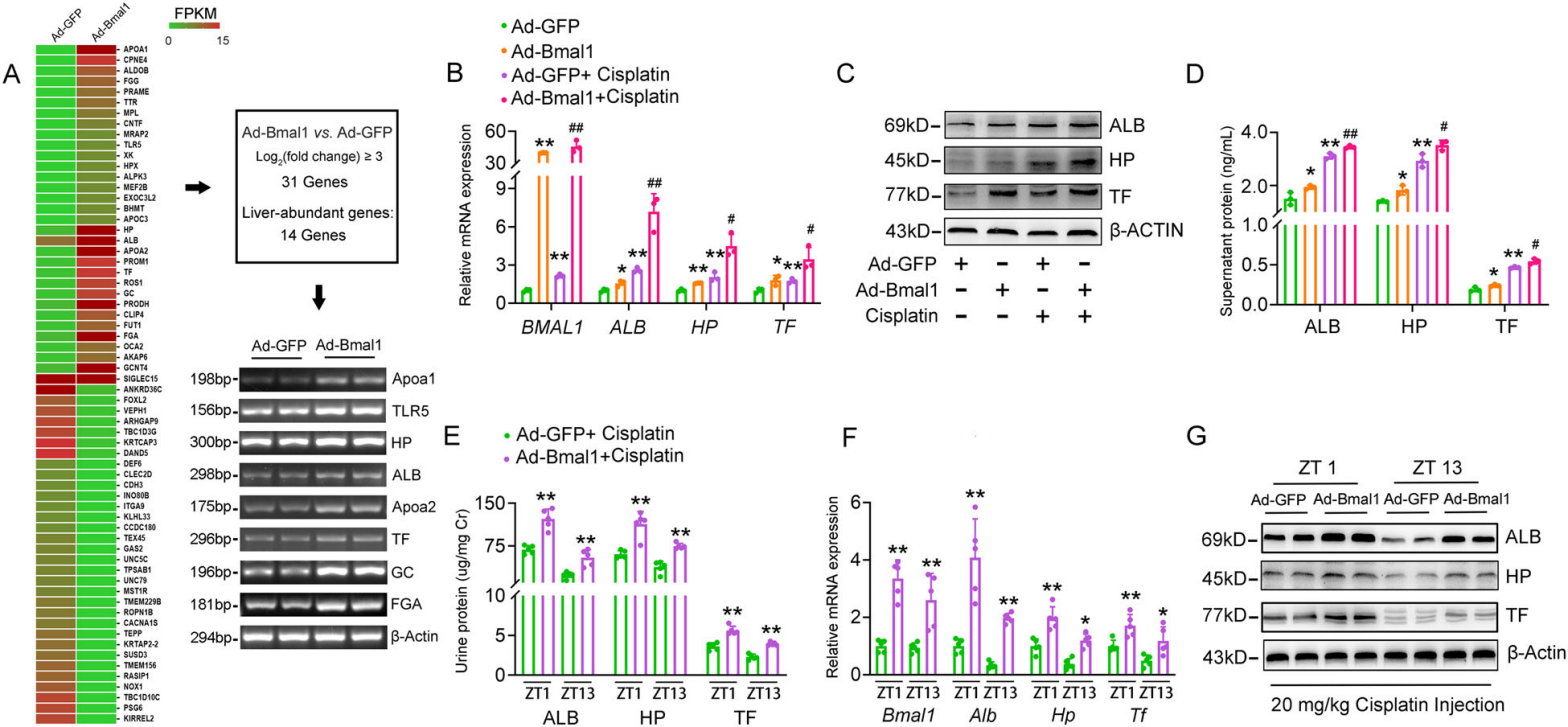
Overexpression of Bmal1 accelerates cisplatin-induced renal hepatization. **a** RNA-Seq analysis in Bmal1-overexpressed HK-2 cells and the representative genes were validated by semi-quantitative RT-PCR analyses. For **b**–**d**, HK-2 cells were treated as described in Fig. S2. **b** RT-qPCR analyses of expression levels of BMAL1, ALB, HP and TF in HK-2 cells. **c** Western blot analyses of expression levels of ALB, HP and TF in HK-2 cells. **d** ELISA analyses of the supernatant levels of ALB, HP and TF. *n* = 3, *P < 0.05 and **P < 0.01 vs. Ad-GFP group, ^#^*P* < 0.05 and ^##^*P* < 0^.^01 vs. Ad-GFP + cisplatin group. For the in vivo analyses, mice were treated as described in Fig. 3. *n* = 5 for each group. **e** Urine levels of Alb, Hp, and Tf. Data were normalized to urine Cr. **f** RT-qPCR analyses of renal expression levels of Bmal1, Alb, Hp and Tf. **g** Western blot analyses of renal expression levels of Alb, Hp and Tf. *P < 0.05 and **P < 0.01 vs. Ad-GFP + cisplatin group. All values are presented as the mean ± SD.

**Fig. 6:**
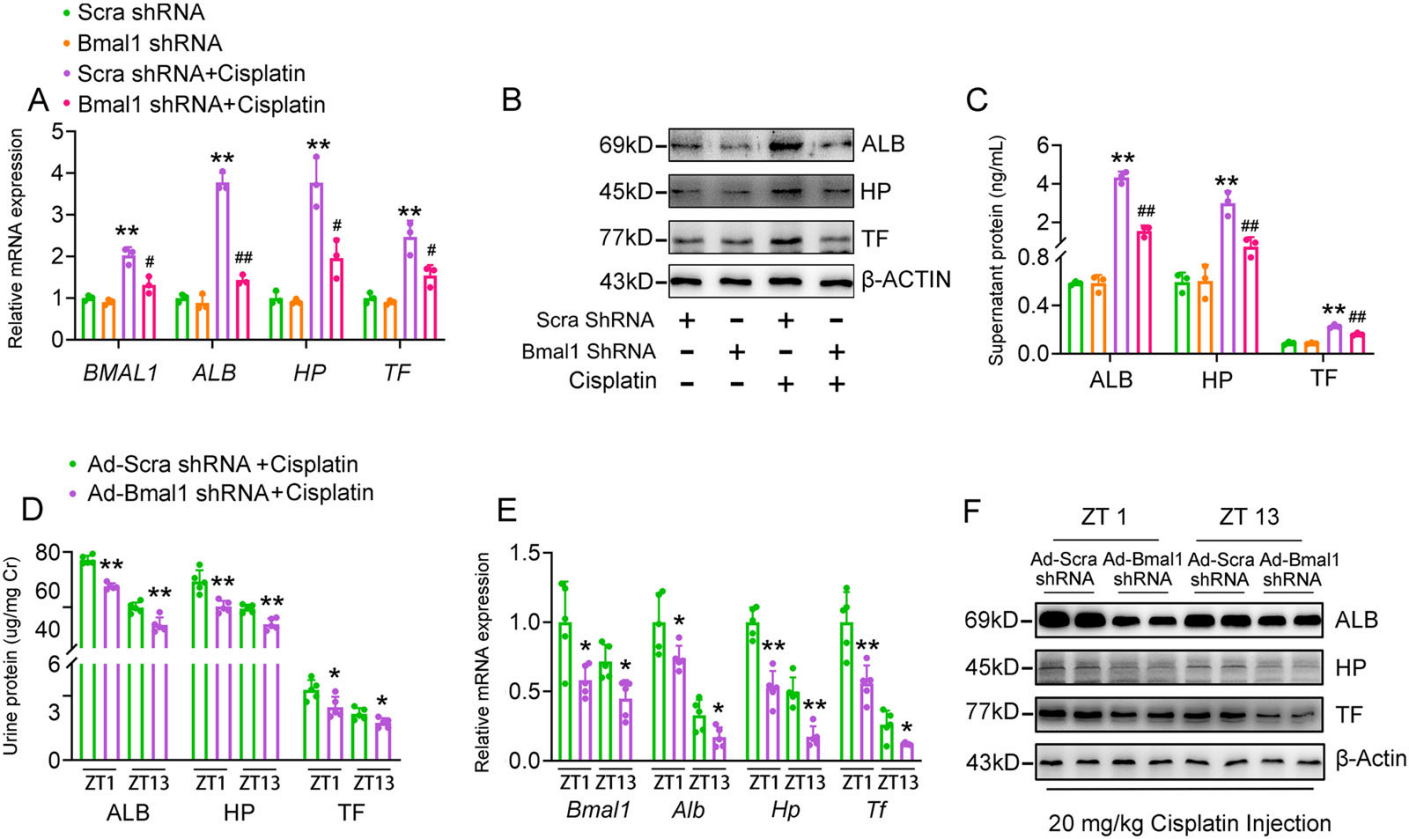
Knockdown of Bmal1 alleviates cisplatin-induced renal hepatization. HK-2 cells were treated as described in Fig. S5. **a** RT-qPCR analyses of expression levels of BMAL1, ALB, HP and TF in HK-2 cells. **b** Western blot analyses of expression levels of ALB, HP and TF in HK-2 cells. **c** ELISA analyses of the supernatant levels of ALB, HP and TF. *n* = 3, *P < 0.05 and **P < 0.01 *vs*. scramble shRNA group, ^#^*P* < 0.05 and ^##^*P* < 0.01 vs. scramble shRNA + cisplatin group. For the in vivo analyses, mice were treated as described in Fig. S5. *n* = 5 for each group. **d** Urine levels of Alb, Hp, and Tf. Data were normalized to urine Cr. **e** RT-qPCR analyses of renal expression levels of Bmal1, Alb, Hp and Tf. **f** Western blot analyses of renal expression levels of Alb, Hp and Tf. *P < 0.05 and **P < 0.01 vs. Ad-scramble shRNA + cisplatin group. All values are presented as the mean ± SD.

Since Bmal1 facilitates cisplatin-induced renal injury, we wanted to identify the downstream effectors that mediate this progress. To address this concern, we peroformed transcriptional profiling in HK-2 cells with Bmal1 overexpression. As shown in Fig. 5A and Fig. S7A, forced expression of Bmal1 in HK-2 cells induced expression levels of 31 genes when cut-off threshold was set as >8-fold. Among these, 14 genes were recognized as liver-specific enriched genes, implicating that renal hepatization occurred in response to Bmal1 overexpression. The expression of albumin (ALB), haptoglobin (HP), and transferrin (TF), which were typical mark genes involved in hepatization, was confirmed by RT-PCR analyses. Also, Bmal1 overexpression increased the expression of hepatization-associated genes both at transcriptional and translation levels in cisplatin-treated HK-2 cells (Fig. 5B, C and Fig. S7B, C). Functionally, ELISA analyses indicated that overexpression of Bmal1 stimulated the secretion of ALB, HP, and TF in vehicle- or cisplatin-treated cells (Fig. 5D). Coincidence with the in vitro results, nephron-specific overexpression of Bmal1 promoted cisplatin-induced expression and secretion of these hepatokines at all the examined time points (Fig. 5e–g, Fig. S7D, E and Supplementary Table 4). To confirm the essential role of Bmal1 in cisplatin-induced renal hepatization, we knocked down Bmal1 expression both in vitro and in vivo. The overall findings were opposite to those obtained in the overexpression system, as demonstrated by the decreased expression and secretion of hepatokines even when cisplatin was consistently present in all the settings (Fig. 6a–f, Fig. S7F–I, Supplementary Table 5). In addition, overexpression of Per2 exhibited modest impact on the mRNA expression and secretion of renal hepatization-associated genes, including ALB, HP, and TF (Fig. S7J and K, *P* = 0.2765 for ALB, *P* = 0.2090, for HP, *P* = 0.4484 for TF, *P* = 0.7461 for ALB, *P* = 0.4848, for HP, *P* = 0.9639 for TF).

### Bmal1 activates transcription of ALB, HP, and TF through direct promoter occupancy

**Fig. 7:**
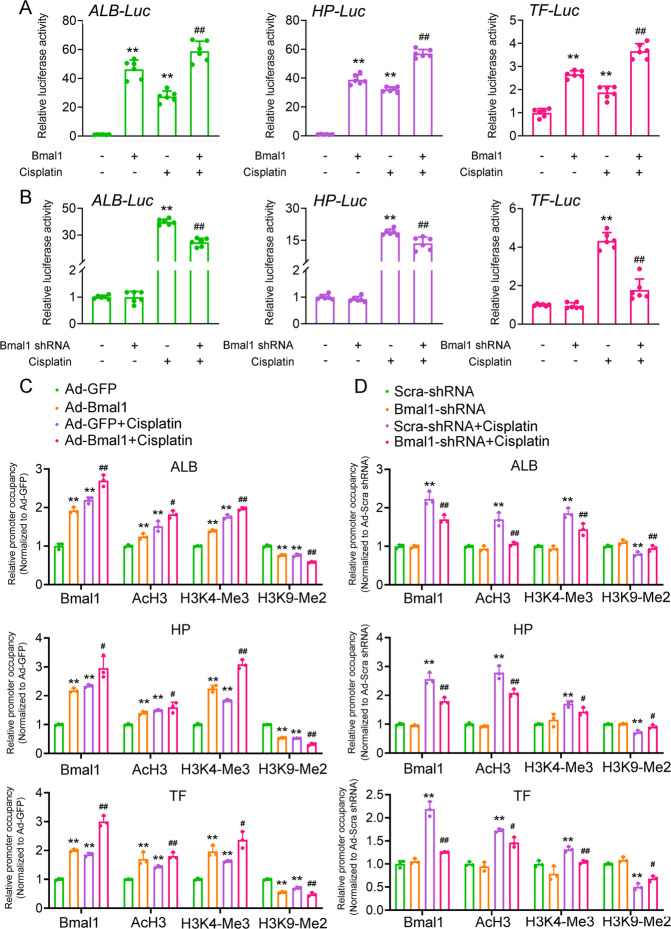
Bmal1 activates transcription of *Alb*, *Hp*, and *Tf* through direct promoter occupancy. **a** Reporter gene assays in HK-2 cells transfected with indicated plasmids for 24 h, and then treated with 20 μM cisplatin or vehicle for another 24 h. *n* = 6, *P < 0.05 and **P < 0.01 vs. vector group, ^##^*P* < 0.01 vs. Bmal1 overexpression or cisplatin alone group. **b** Reporter gene assays in HK-2 cells with Bmal1 knockdown. *n* = 6, **P < 0.01 vs. scramble shRNA group, ^##^*P* < 0.01 vs. scramble shRNA + cisplatin group. **c** ChIP assays with indicated antibodies in HK-2 cells similarly treated as described in Fig. S2. The enrichments were quantified by RT-qPCR analysis. *n* = 3, **P < 0.01 vs. Ad-GFP group, ^#^*P* < 0.05 and ^##^*P* < 0.01 vs. Ad-GFP + cisplatin group. **d** ChIP assays with indicated antibodies in HK-2 cells similarly treated as described in Fig. S5. The enrichments were quantified by RT-qPCR analysis. *n* = 3, **P < 0.01 vs. scramble shRNA group, ^#^*P* < 0.05 and ^##^*P* < 0.01 *vs*. scramble shRNA + cisplatin group. All values are presented as the mean ± SD.

To determine whether Bmal1 induces the expression of renal hepatization-associated genes at the transcriptional level, we constructed luciferase reporter plasmids containing the proximal regions of human ALB, HP, and TF promoters. Either transient transfection of Bmal1 or cisplatin treatment alone was already able to increase the luciferase activities of these reporter plasmids (Fig. 7a). A combination of these two manipulations showed a synergetic effect. In contrast, knockdown of Bmal1 antagonized the activation of these promoters induced by cisplatin (Fig. 7b). Besides, bioinformatics analysis reveals the several E-box motifs present on these promoter regions and our chromatin immunoprecipitation (ChIP) assays confirmed that Bmal1 recruited to the E-box regions of *ALB*, *HP*, and *TF* promoters in response to cisplatin stimulation. Furthermore, histone modification is known to be associated with gene transcriptional activity. Acetylated Histone 3 (AcH3) and histone H3 trimethylated at lysine 4 (H3K4-me3) are hallmarks of actively transcribed genes, whereas histone H3 dimethylated at lysine 9 (H3K9-me2) is found in heterochromatin and silenced genes. We found that either Bmal1 overexpression or cisplatin treatment resulted in a remarkable increase in AcH3 and H3K4me3 (activation) levels accompanied by a reduction of H3K9me2 (repression) levels on the proximal regions of all three gene promoters (Fig. 7c). The knockdown of Bmal1, in turn, caused converse results (Fig. 7d).

### Kinases activation mediates cisplatin-induced Bmal1 expression

**Fig. 8:**
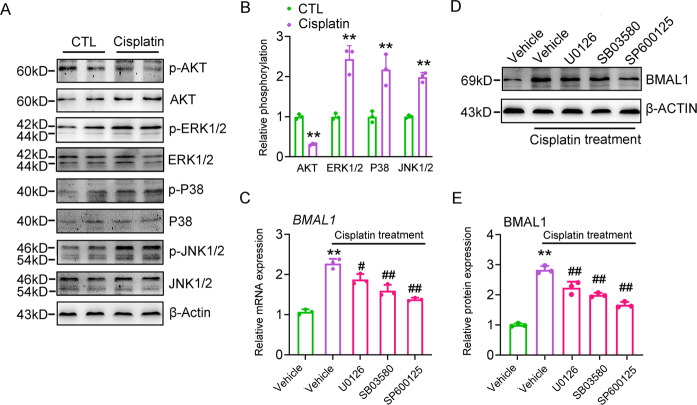
Kinases activation mediates cisplatin-induced Bmal1 expression. **a** Western blot analyses of AKT, ERK1/2, P38, and JNK phosphorylation in HK-2 cells treated with 20 μM cisplatin for 24 h. **b** Quantitative analyses of **a**. *n* = 3, **P < 0.01 vs. CTL group. **c**, **d** RT-qPCR and western blot analyses of BMAL1 expression in HK-2 cells treated with 20 μM cisplatin alone or in combinations of various signaling pathway inhibitors for 24 h. *n* = 3, **P < 0.01 vs. vehicle group, ^#^*P* < 0.05 and ^##^*P* < 0.01 vs. cisplatin alone group. **e** Quantitative analysis of panel **d**. *n* = 3, **P < 0.01 vs. vehicle group, ^##^*P* < 0.01 cisplatin + inhibitor vs. cisplatin alone group. All values are presented as the mean ± SD. Concentrations: U0126, 10 μM; SB203580, 10 μM; SP600125, 10 μM.

To identify potential downstream mediators in cisplatin-induced Bmal1 expression, we examined the phosphorylation of chief factors, such as MAPKs and AKT, which have been shown to be responsive for the cisplatin stimulation. Cisplatin increased the phosphorylation levels of ERK1/2, P38, and JNK1/2, while reducing the AKT phosphorylation levels (Fig. 8a, b). More importantly, U0126 (ERK1/2 inhibitor), SB203580 (P38 MAPK inhibitor), and SP600125 (JNK1/2 inhibitor) almost abolished the cisplatin-induced BMAL1 transcription and translation. Among which, SP600125 demonstrated the most effective suppression action (Fig. 8c–e). These data suggest that ERK/P38/JNK, MAPKs may mediate the induction of cisplatin on BMAL1 expression.

## Discussion

Cisplatin is widely used as a functional therapeutic reagent in the treatments of tumors^27^. However, the accompanied nephrotoxicity significantly restricts its clinical application^28^. Previous studies have indicated that using the concept of chronotherapy, administration at specific times of the day, may potentially minimize its toxicity. For example, clinical investigations revealed that toxic side-effects of cisplatin, including nephrotoxicity, were alleviated when administered in the afternoon^14^. Animal experiments suggested that less cisplatin-DNA adducts were found in the kidney when cisplatin was injected at the active period (ZT12)^29^. Not surprisingly, cisplatin-induced nephrotoxicity actually can be divided into multiple stages. For example, cisplatin should first be incorporated into renal cells by organic cation transporter 2 (OCT2), and then forms DNA adducts, resulting in the activation of apoptotic signals and the renal cell death. More importantly, the role of different clock genes in the individual stage has been revealed. During the cisplatin uptake controlled by OCT2, it has been shown that OCT2 is rhythmically expressed and its rhythmicity is orchestrated by the Clock-PPARα axis^24^. For the clearance of DNA adducts, XPA, a key component in the NER (nucleotide excision repair) process, plays an important role and this factor also shows a robust oscillation under the control of Cry family members^30^. However, it is still unknown whether the physiological output (renal cell apoptosis and hepatization) is also regulated by a certain clock gene. Filling this blank is the aim of our study and we provided solid evidence to show that Bmal1 is a critical factor involved in this process. Based on previous and our studies, we believe that indeed, cisplatin-induced nephrotoxicity is the final outcome of comprehensive interactions of various clock components and each stage of cisplatin nephrotoxicity is precisely controlled by a specific clock gene. Also, the phosphorylation of MAPKs is extensively involved in the regulation of circadian clock^31,32^. Given that MAPK signaling pathways have been identified to contribute to the cisplatin-induced nephrotoxicity^33–35^, our findings strongly suggest that various MAPKs may mediate the induction of Bmal1 expression by cisplatin. Indeed, we found that cisplatin-triggered phosphorylation of JNK1/2, ERK1/2 and p38 MAPK mediated its action on the Bmal1 expression (Fig. 8), which revealed that Bmal1 is an authentic clock gene regulated by cisplatin.

As mentioned above, the first step of cisplatin, the absorption and accumulation of cisplatin in renal cells exhibit a circadian oscillation as well. This process occurs more robustly during the rest period than that in the active period^24^, leading to the difference in the in situ functional drug doses when cisplatin is administered at various time points. Such chronopharmacokinetics of cisplatin is an important factor contributing to the final outcome of its pharmacological function. Therefore, if we injected cisplatin into mice at different time points, the changes in the circadian phases or amplitudes of clock genes we observe could be induced by cisplatin itself, as we speculate, but could also be induced by the chronopharmacokinetics of cisplatin. Taken these variables into consideration, we injected cisplatin into mice only at ZT1 and ZT13, respectively, in our original experimental design. For these two time points, the expression of OCT2, which is a key factor responsive for the cisplatin uptake, is similar, guaranteeing that the first step of cisplatin pharmacological actions (absorption) is comparable^24^. Only when we fix this variable, can we conclude that the observed difference in the expression patterns of clock genes is purely due to the cisplatin itself.

As a key component in the circadian clock machinery, Bmal1 has been recognized to play an important role in the sensitivity of anti-tumor drugs. For example, Bmal1-deficient fibroblasts exhibit reduced sensitivity to DNA-damaging anticancer drugs, such as etoposide and daunorubicin, while Bmal1 knockout mutation in the breast cancer cell line MCF10A sensitizes to cisplatin-induced apoptosis^36,37^. In contrast, Bmal1 overexpression increases the sensitivity to oxaliplatin in colorectal cancer^38^. All these findings revealed a causal relationship between Bmal1 and the efficiency of anti-tumor drugs. In our study, we examined the effect of cisplatin on Bmal1 expression in the kidney tissues/cultured cells, and found that overexpression of Bmal1 aggravated the cisplatin-induced renal injury in vivo and accelerated cell apoptosis in vitro (Figs. 3 and 4, Figs. S2 and S5), indicating that Bmal1 is sufficient to increase the sensitivity to cisplatin in the kidney leading to the renal injury. On the other hand, we also noticed that nephron-specific knockdown of Bmal1 alleviated the cisplatin-induced renal injury at two treatment time points. Moreover, when Bmal1 expression gets further decreased by the shRNA oligonucleotides at ZT13, the pathological changes induced by cisplatin treatment were almost completely diminished when compared to the control mice (Figs. 1 and 4). These results indicate that Bmal1 responses to cisplatin in a clock-dependent manner, and aggravates cisplatin-induced renal injury independent of clock factors (Supplementary Tables 2–5). Notably, Bmal1 is also known to have non-clock functions. For example, the germline *Bmal1* knockout is associated with early aging, while the inducible *Bmal1* knockout mice exhibit no gross effect^39^. Therefore, more studies are needed to pursue the clock-dependent or clock-independent role of Bmal1 in mediating the cisplatin-induced renal injury by using *Per1/2* double knockout mice or HK-2 cells.

Since we identified Bmal1 as a pivotal mediator in cisplatin-induced renal injury, and the therapeutic intervention targeting Bmal1 in the kidney may be a promising strategy to minimize the toxic side-effects of cisplatin in its clinical applications, this would raise a serious concern that the anti-tumor effect of cisplatin may be reduced while decreasing the toxic side-effect of cisplatin with Bmal1 manipulation. It should be noted that numerous studies have revealed the role of Bmal1 in tumorigenesis and they consistently pointed out that Bmal1 rhythmicity is blunted in the tumor tissues (the amplitude of Bmal1 oscillation is dampened)^40^. In this sense, the physiological importance of Bmal1 rhythmicity in the tumors can be largely ignored. On the other hand, the actions of Bmal1 in tumor development are tissue-specific and diverse^41^. For example, Bmal1 is an oncogene in breast cancer^42^, while it acts as a tumor suppressor in tongue squamous cell carcinoma^43^. Therefore, the anti-tumor effect of cisplatin may be still preserved when we manipulate the local diurnal expression of Bmal1 in the kidney to avoid the toxic side-effect of cisplatin.

For decades, it was believed that proteins with molecular weight <40 kDa in the urine get derived from glomeruli filtration that would escape from the uptake process in the renal tubular system^44^. In contrast, we examined increased urine compositions of proteins with a molecular weight of more than 40 kDa in certain chronic and acute kidney diseases. These proteins include but not limited to ALB, HP, TF, C3, AHSG, soluble IgA, and IgM, which are liver-specific abundant proteins produced and secreted by injured proximal tubular epithelial cells^45–47^. In the phenomenon of renal hepatization, the injured kidney develops certain features of hepatic phenotypes^48^. As a characteristic of the side effects of cisplatin, patients treated with cisplatin also have renal hepatization. For example, cisplatin increases the secretion of ALB into urine of patients suffered from cancers^49^. Animal experiments also demonstrated that cisplatin increases the urine concentrations of ALB, HP, and TF^47,50,51^. These observations suggest the renal hepatization occurs in parallel with the cisplatin-induced renal injury. Unfortunately, the molecular mechanism through which renal injury signals trigger the hepatization is still unclear. In our present study, we performed RNA-seq analyses and identified downstream effectors of Bmal1 in HK-2 cells (a human proximal tubular epithelial cell line). Our results revealed that forced expression of Bmal1 in HK-2 cells induced the renal hepatization process, and we recognized 45.2% of up-regulated genes as liver-specific genes. In parallel, low-grade cell apoptosis has existed in Bmal1-overexpressed HK-2 cells. A combination of Bmal1 overexpression and cisplatin treatment showed a synergetic effect on the hepatization process (Fig. 5). Conversely, not only the knockdown of Bmal1 with shRNA oligonucleotides did not alter the basal expression of these hepatization-associated genes, despite fundamentally, Bmal1 adequately promotes the activation of hepatization. Besides, to elucidate the role of E-box in the mediation of Bmal1-induced transcription of the hepatization-associated gene, we performed luciferase reporter assays and ChIP experiments. Our study indicated that Bmal1 recruits to the E-box motifs of proximal promoter regions of these genes, and functionally alters the chromatin structure into an active state, finally turning on their transcription. For the first time, we identified a significant regulator in triggering cisplatin-induced hepatization and provided an exquisite mechanism to explain how this regulator modulates hepatization at the transcriptional level.

In conclusion, our findings suggest that Bmal1 is a fundamental mediator to induce renal injury, and hepatization in response to cisplatin treatment. The therapeutic intervention targeting Bmal1 in the kidney may be a promising strategy to minimize the toxic effects of cisplatin in its clinical applications.

## Materials and methods

### Animals

All animal procedures in this investigation conform to the Guide for the Care and Use of Laboratory Animals published by the US National Institutes of Health (NIH publication No. 85–23, revised 1996) and the approved regulations set by the Laboratory Animal Care Committee at Nanjing University of Chinese Medicine (Permit number: ACU170607). Male C57BL/6J mice were maintained in a 12 h:12 h LD cycle and in a temperature- and humidity-controlled environment. To induce renal injury, mice were received intraperitoneal injection of cisplatin at a dose of 20 mg/kg body weight or equivalent volume of 0.9% saline solution at the time point ZT1 and ZT13, respectively. To artificially manipulate Bmal1 expression in the kidney, we transduced adenoviruses encoding either CDS domain of Bmal1 or Bmal1 shRNA, or their negative controls into mice at a dose of 1 × 10^10^ plaque-forming units (PFU) per mouse through tail-vein injection. Detailed sequences for the shRNA oligonucleotide sequences were listed in Supplementary Table 6. Three days after the transduction, all mice were received a single injection of either cisplatin (20 mg/kg) or 0.9% saline solution at indicated time points. In all, 72 h later, these mice were sacrificed by cervical dislocation to collect sera and kidney samples. Urine was collected before sacrifice.

### Cell culture and transfection

Human renal tubular epithelial HK-2 cells (ATCC, Manassas, VA, USA) were cultured at 37 °C and 5% CO_2_ in RPMI-1640 medium supplemented with 10% fetal bovine serum (FBS) and 1% antibiotics (penicillin and streptomycin). To knock down Bmal1 in HK-2 cells, these cells were transfected with either Bmal1 shRNA or scramble shRNA for 24 h followed by 24-h cisplatin treatment. We performed transfection by using Lipofectamine 3000 reagent (Invitrogen, Carlsbad, CA, USA) according to the manufacturer’s instructions.

### RT-qPCR and western blot analyses

Total RNA was isolated using Trizol reagent (Invitrogen, Carlsbad, CA, USA). For mRNA detection, 1 μg total RNA was reverse-transcribed into cDNA. Real-time PCR amplification was performed using SYBR premix Ex Taq (Vazyme, Nanjing, Jiangsu, China) and the LightCycler® 480 System (Roche, Basal, Switzerland). The primers for both human and mouse *β-ACTIN* were included for normalization. A complete list of PCR primers is shown in Supplementary Table 6. For protein analysis, kidney tissues were homogenized, and cells were lysed in ice-cold RIPA buffer. The protein concentration was quantified with a BCA protein quantification kit (Beyotime, Shanghai, China). Equal amounts of protein were loaded and separated by 10% SDS-PAGE, and then transferred onto PVDF membranes (Millipore, Bedford, MA, USA). The membranes were incubated overnight with indicated primary antibodies. HRP-conjugated secondary antibodies were then applied to bind and visualize the primary antibodies. Quantitative analysis was performed by AlphaEaseFC software (version 3.1.2 Alpha Innotech Corporation, CA, USA). The anti-BMAL1 antibody was purchased from Abcam (Cat. No. ab3350, 1:1000 dilution). The antibodies against β-ACTIN (Cat. No. AP0060, 1:1000 dilution), ALB (Cat. No. BS6520, 1:1000 dilution), HP (Cat. No. BS90611, 1:1000 dilution), TF (Cat. No. BS7016, 1:1000 dilution), BAX (Cat. No. BS1030, 1:1000 dilution), BCL-2 (Cat. No. BS70205, 1:1000 dilution), and cleaved-Caspase-3 (Cat. No. BS7073, 1:1000 dilution) were obtained from Biogot Technology (Nanjing, Jiangsu, China). The antibodies against ERK1/2 (Cat. No. 9102 s, 1:1000 dilution), phospho-ERK1/2 (Thr202/Tyr204, Cat. No. 4377 s, 1:1000 dilution), JNK (Cat. No. 9252 s, 1:1000 dilution), phospho-JNK1/2 (Thr183/Tyr185, Cat. No. 4668 T, 1:1000 dilution), AKT (Cat. No. 9252 s, 1:1000 dilution), phospho-AKT (Ser 473, Cat. No. 4060, 1:1000 dilution), P38 (Cat. No. 9212 s, 1:1000 dilution), Kim-1 (Cat. No. 14971 s, 1:1000 dilution) and NGAL (Cat. No. 44058, 1:1000 dilution) were purchased from Cell Signaling Technology (Danvers, MA, USA). The anti-PER2 antibody was purchased from Proteintech (Wuhan, Hubei, China). The anti-phospho-P38 (Tyr182, Cat. No. 11253, 1:1000 dilution) was purchased from SABiosciences (Frederick, MD, USA). For all the Western blot images throughout our study, a representative image was shown from at least three separate experiments.

### High-throughput RNA sequencing

To identify downstream genes mediating Bmal1-induced renal injury and hepatization, HK-2 cells were infected by recombinant adenoviruses carrying either Bmal1 CDS domain or GFP for 48 h. Total RNA was isolated for the construction of RNA-seq libraries. The quality of the RNA libraries was evaluated using the Agilent 2200 TapeStation (Agilent Technologies, USA). Library sequencing was performed on a HiSeq 3000 sequencing platform (Illumina Company, USA) by Novogene Corp., China. Fragments Per Kilobase of transcript per Million mapped reads (FPKM) were calculated for additional statistics. All the reads were mapped to the human genome (GRCh38/hg38).

### Serological and urine analyses

Blood samples were collected in non-heparinized tubes and centrifuged at 4000 rpm for 10 min at 4 °C. BUN and serum Cr levels were determined spectrophotometrically, and urine Cr, ALB, HP and TF levels were quantified by ELISA, using commercial kits purchased from Nanjing Jiancheng Bioengineering Institute (Nanjing, Jiangsu, China).

### Histological and IHC analyses

Mouse kidneys were isolated, fixed in 4% paraformaldehyde solution for 24 h, processed for paraffin embedding, and cut into 4 μm transverse sections, deparaffinized and rehydrated using standard technique. The sections were stained with H&E and PAS in order to detect the following histological changes in the kidney: (1) proximal renal tubular dilation, (2) hyaline cast, (3) epithelial cell detachment. For IHC analysis, the sections were incubated with indicated primary antibodies at 4 °C overnight for the later immunostaining by using diaminobenzidine (DAB). Non-immune IgG was used as a negative control. The sections were photographed with a Nikon fluorescence microscope (×400 magnification, ECLIPSE, Ts2R-FL, Tokyo, Japan).

### TUNEL staining

As described above, the kidney paraffin sections were made, and were undergone dewaxing and dehydration. Proteinase K was added to cover the sample area and incubated for 30 min at room temperature. The sections were then blocked for 10 min, followed by a 60-min incubation with the TUNEL reaction mixture. After a 30-min Conversion-agent-POD incubation, DAB chromogen was used for visualization of apoptotic cells. The nuclei were stained with hematoxylin. All the sections were photographed with a Nikon fluorescence microscope (×400 magnification, ECLIPSE, Ts2R-FL, Tokyo, Japan). On the other hand, apoptotic cultured cells were detected by using a TUNEL BrightRed Apoptosis Detection Kit (Vazyme, Nanjing, Jiangsu, China) according to the manufacturer’s instructions. Signals were visualized using a Nikon fluorescence microscope (×400 magnification, ECLIPSE, Ts2R-FL, Tokyo, Japan), and the average ratios between TUNEL-positive (pink) and total DAPI-stained nuclei (blue) were calculated for statistical analyses.

### Flow cytometry analyses

HK-2 cells were detached by 0.25% Trypsin-EDTA digestion and collected by centrifugation at 1000 rpm for 5 min. The cells were re-suspended at the density of 1 × 10^6^ cells/mL and stained with Annexin V-APC/7AAD Apoptosis Detection Kit (Keygentec, Nanjing, Jiangsu, China) according to the manufacturer’s instructions. Finally, the positive signals of apoptotic cells were analyzed with FACSCalibur flow cytometer (Becton Dickinson, San Diego, CA, USA).

### Luciferase reporter assays

The promoter regions flanking *ALB* (−410 to −71 bp), *HP* (−405 to +5 bp) and *TF* (−376 to +89 bp) were amplified from human genomic DNA by the PCR method. Detailed primer sequences were listed in Supplementary Table 6. The amplified fragments were validated by sequencing and cloned into a PGL3-basic vector using cutting sites of KpnI and XhoI restriction enzymes. For luciferase reporter assays, HK-2 cells were co-transfected with 200 ng reporter plasmids and 800 ng expression constructs encoding BMAL1 CDS region or shRNA against BMAL1, and 5 ng of control pRL-TK plasmids as an internal control. 24 h later, cells were treated with or without cisplatin for another 24 h. Equal amounts of DNA were used for all the transfections by adding appropriate empty vector DNA. Relative luciferase activities (firefly luciferase values were normalized to renilla luciferase values) were determined 48 h following transfection using the Luciferase system (Promega, Madison, WI, USA).

### ChIP assays

ChIP analyses were performed in Bmal1-overexpression and Bmal1-knockdown HK-2 cells. In brief, these cells were cross-linked with 1% formaldehyde at room temperature for 7 min and lysed with ChIP lysis buffer. The cell lysates were sonicated to shear the chromatin and immunoprecipitated with antibodies against BMAL1, AcH3, H3K4-me3 (Cat. No. ab1012, Abcam, Cambridge, MA, USA) and H3K9-me2 (Cat. No. ab1220, Abcam, Cambridge, MA, USA) in the presence of BSA and salmon sperm DNA. The immunoprecipitants were isolated using protein G-agarose beads. After reversing the cross-links by incubation at 65 °C overnight, the pulled-down DNA was purified and was analyzed by RT-qPCR using primers flanking the proximal E-box domain on the human promoters of *ALB*, *HP*, and *TF* genes. Detailed sequences were listed in Supplementary Table 6.

### Statistical analyses

Statistical analyses were calculated by using GraphPad Prism software (version 8.0.2 OriginLab Corporation, MA, USA). The data are presented as the means ± SD (standard deviation). All data were analyzed using one-way or two-way ANOVA followed by Bonferroni’s *posthoc* test. Detailed *F* and *P* values were presented in Supplementary Tables 1, and 2–5. A *P*-value of <0.05 was considered to be statistically significant.

## Supplementary information

Supplementary Tables

Supplementary Figure Legends

Supplementary Figure 1

Supplementary Figure 2

Supplementary Figure 3

Supplementary Figure 4

Supplementary Figure 5

Supplementary Figure 6

Supplementary Figure 7
