# Supplementary material for: The circadian clock gene Bmal1 facilitates cisplatin-induced renal injury and hepatization
Source: Cell Death Dis. 2020 Jun 10;11(6):446. doi: 10.1038/s41419-020-2655-1 (PMC7287064; doi:10.1038/s41419-020-2655-1)
Supplement: Supplementary file 1 — Supplementary Tables [file 41419_2020_2655_MOESM1_ESM.docx]

**Supplementary Tables**

**Supplementary Table 1** *F* and *P* values of two-way ANOVA analyses of the BUN, Serum Cr, the relative mRNA and protein expression levels in mice treated as in Fig. 1.

| Parameters | Cisplatin | | ZT | | Cisplatin × ZT | |
| --- | --- | --- | --- | --- | --- | --- |
|  | *F* Values | *P* Values | *F* Values | *P* Values | *F* Values | *P* Values |
| BUN | 14.97 | 0.0047 | 158 | < 0.0001 | 23.06 | 0.0014 |
| Serum Cr | 17.99 | 0.0028 | 17.46 | 0.0031 | 12.91 | 0.0071 |
| **mRNAs** |  |  |  |  |  |  |
| *Kim-1* | 11.88 | 0.0087 | 24.97 | 0.0011 | 11.92 | 0.0087 |
| *Ngal* | 146.3 | < 0.0001 | 62.64 | < 0.001 | 144.5 | < 0.0001 |
| **Proteins** |  |  |  |  |  |  |
| Kim-1 | 48.76 | < 0.0001 | 1100 | < 0.0001 | 131.1 | < 0.0001 |
| Ngal | 75.3 | < 0.0001 | 370.2 | < 0.0001 | 28.46 | 0.0003 |

**Supplementary Table 2** *F* and *P* values of two-way ANOVA analyses of the BUN, Serum Cr, the relative mRNA and protein expression levels in mice treated as in Fig. 3 and Fig. S3.

| Parameters | Ad-Bmal1 | | ZT | | Ad-Bmal1 × ZT | |
| --- | --- | --- | --- | --- | --- | --- |
|  | *F* Values | *P* Values | *F* Values | *P* Values | *F* Values | *P* Values |
| BUN | 10.64 | 0.0115 | 13.32 | 0.0065 | 1.132 | 0.3185 |
| Serum Cr | 44.15 | 0.0002 | 20.22 | 0.002 | 0.3277 | 0.5828 |
| **mRNAs** |  |  |  |  |  |  |
| *Kim-1* | 16.36 | 0.0037 | 31.39 | 0.0005 | 1.597 | 0.2419 |
| *Ngal* | 20.24 | 0.002 | 12.27 | 0.008 | 0.7612 | 0.4084 |
| **Proteins** |  |  |  |  |  |  |
| Kim-1 | 680.1 | < 0.0001 | 351.5 | < 0.0001 | 4.798 | 0.0599 |
| Ngal | 514.5 | < 0.0001 | 572 | < 0.0001 | 5.22 | 0.0517 |

**Supplementary Table 3** *F* and *P* values of two-way ANOVA analyses of the BUN, Serum Cr, the relative mRNA and protein expression levels in mice treated as in Fig. 4 and Fig. S6.

| Parameters | Ad-Bmal1 shRNA | | ZT | | Ad-Bmal1 shRNA × ZT | |
| --- | --- | --- | --- | --- | --- | --- |
|  | *F* Values | *P* Values | *F* Values | *P* Values | *F* Values | *P* Values |
| BUN | 18.27 | 0.0027 | 15.92 | 0.004 | 0.0416 | 0.8434 |
| Serum Cr | 75.32 | < 0.0001 | 9.535 | 0.0149 | 5.115 | 0.0536 |
| **mRNAs** |  |  |  |  |  |  |
| *Kim-1* | 32.53 | 0.0005 | 8.92 | 0.0174 | 1.848 | 0.2111 |
| *Ngal* | 63.8 | < 0.0001 | 14.24 | 0.0054 | 2.819 | 0.1317 |
| **Proteins** |  |  |  |  |  |  |
| Kim-1 | 102.1 | < 0.0001 | 53.84 | < 0.0001 | 0.6326 | 0.4493 |
| Ngal | 52.16 | < 0.0001 | 359.7 | < 0.0001 | 3.226 | 0.1102 |

**Supplementary Table 4** *F* and *P* values of two-way ANOVA analyses of the relative mRNA and protein expression and urine levels of hepatization-related genes in mice treated as in Fig. 5 and Fig. S7.

| Parameters | Ad-Bmal1 | | ZT | | Ad-Bmal1× ZT | |
| --- | --- | --- | --- | --- | --- | --- |
|  | *F* Values | *P* Values | *F* Values | *P* Values | *F* Values | *P* Values |
| **mRNAs** | | | | | | |
| *Alb* | 18.56 | 0.0026 | 62.43 | < 0.0001 | 5.002 | 0.0557 |
| *Hp* | 38.14 | 0.0003 | 42.39 | 0.0002 | 0.4188 | 0.5356 |
| *Tf* | 12.17 | 0.0082 | 20.69 | 0.0019 | 0.0032 | 0.926 |
| **Proteins** |  |  |  |  |  |  |
| Alb | 2323 | < 0.0001 | 669.1 | < 0.0001 | 0.6114 | 0.4568 |
| Hp | 97.8 | < 0.0001 | 354.1 | < 0.0001 | 0.6467 | 0.4445 |
| Tf | 872.4 | < 0.0001 | 410.2 | < 0.0001 | 0.1838 | 0.6795 |
| **Secretion** |  |  |  |  |  |  |
| Alb | 98.26 | < 0.0001 | 68.79 | < 0.0001 | 3.94 | 0.024 |
| Hp | 36.22 | 0.0003 | 51.28 | < 0.0001 | 2.657 | 0.1417 |
| Tf | 47.34 | 0.0001 | 194.3 | < 0.0001 | 0.6758 | 0.4349 |

**Supplementary Table 5** *F* and *P* values of two-way ANOVA analyses of the relative mRNA and protein expression and urine levels of hepatization-related genes in mice treated as in Fig. 6 and Fig. S7.

| Parameters | Ad-Bmal1 shRNA | | ZT | | Ad-Bmal1 shRNA × ZT | |
| --- | --- | --- | --- | --- | --- | --- |
|  | *F* Values | *P* Values | *F* Values | *P* Values | *F* Values | *P* Values |
| **mRNAs** | | | | | | |
| *Alb* | 84.88 | < 0.0001 | 18.65 | 0.0026 | 0.5446 | 0.4816 |
| *Hp* | 102.4 | < 0.0001 | 63.05 | < 0.0001 | 3.228 | 0.1101 |
| *Tf* | 88.11 | < 0.0001 | 24.15 | 0.0012 | 5.799 | 0.0426 |
| **Proteins** |  |  |  |  |  |  |
| Alb | 46.87 | 0000.1 | 143.5 | <0.0001 | 3.301 | 0.1068 |
| Hp | 482.5 | < 0.0001 | 35.36 | 0.0003 | 0.3971 | 0.5462 |
| Tf | 109.2 | < 0.0001 | 103.2 | < 0.0001 | 3.26 | 0.1086 |
| **Secretion** |  |  |  |  |  |  |
| Alb | 353.2 | < 0.0001 | 78.91 | < 0.0001 | 4.067 | 0.0785 |
| Hp | 60.6 | < 0.0001 | 32.56 | 0.0005 | 2.892 | 0.1274 |
| Tf | 26.8 | 0.0008 | 14.7 | 0.005 | 1.184 | 0.3082 |

**Supplementary Table 6** Lists of primer sequences for RT-qPCR analyses, promoter construction, ChIP assays and shRNA oligonucleotides for gene knockdown.

| **List of primers used for RT-qPCR analyses.** | | | | |
| --- | --- | --- | --- | --- |
| **Mouse Genes** | **Forward Primer Sequences (5’-3’)** | | | **Reverse Primer Sequences (5’-3’)** |
| *β-Actin* | AGCCATGTACGTAGCCATCC | | | CTCTCAGCTGTGGTGGTGAA |
| *Bmal1* | TGGAGGGACTCCAGACATTC | | | TGGGACTACTTGATCCTTGG |
| *clock* | CACTCTCACAGCCCCACTGTAC | | | CCCCACAAGCTACAGGAGCAGT |
| *Per1* | AACGGGATGTGTTTCGGGGTGC | | | AGGACCTCCTCTGATTCGGCAG |
| *Per2* | TGATCGAGACGCCTGTGCTCGT | | | CTCCACGGGTTGATGAAGCTGG |
| *Cry1* | AGCGCAGGTGTCGGTTATGAGC | | | ATAGACGCAGCGGATGGTGTCG |
| *Cry2* | TGGGCATCAACCGATGGAG | | | CCCATTCCTTGAACAGCCTTG |
| *Kim-1* | GTGGAAGTAAAGGGGGTGGT | | | TGCCCCTTTAAGTTGTACCG |
| *Ngal* | ACTACAACCAGTTCGCCATG | | | TGATGTTGTCGTCCTTGAGG |
| *Alb* | AATTGGCAACAGACCTGACC | | | CCTCAACAAAATCAGCAGCA |
| *Hp* | GGGAGCTGTTGTCACTCTCC | | | TCACATTCGGGGAGTTTCTC |
| *Tf* | GCGCATTCAAGTGTCTGAAA | | | GAGCCACAACAGCATGAGAA |
| **Human Genes** | **Forward Primer Sequences (5’-3’)** | | | **Reverse Primer Sequences (5’-3’)** |
| *β-ACTIN* | CACCCACACTGTGCCCATCTACGA | | | CAGCGGAACCGCTCATTGCCAATGG |
| *BMAL1* | AGAGGTGCCACCAATCCATAC | | | CCTCGGTCACATCCTACGACA |
| *CLOCK* | AAGTTAGGGCTGAAAGACGACG | | | GAACTCCGAGAAGAGGCAGAAG |
| *PER1* | CTGAGGAGGCCGAGAGGAAAGAA | | | AGGAGGAGGAGGCACATTTACGC |
| *PER2* | CGTGCCAAGCAGTTGACTTA | | | CAGCAAGGCTCAACAAATCA |
| *CRY1* | CAATGGTGAACCATGCTGAG | | | TCCACTGCTGCTACAACCTG |
| *CRY2* | CCAAGAGGGAAGGGCAGGGTAGAG | | | AGGATTTGAGGCACTGTTCCGAGG |
| *KIM-1* | ACCTTTGTTCCTCCAATGCC | | | ACGGTGTCATTCCCATCTGT |
| *NGAL* | ATGTCACCTCCGTCCTGTTT | | | AGTCAGCTCCTTGGTTCTCC |
| *BAX* | AAGAAGCTGAGCGAGTGTCT | | | CCAATGTCCAGCCCATGATG |
| *BCL-2* | TTCTTTGAGTTCGGTGGGGT | | | CTTCAGAGACAGCCAGGAGA |
| *ALB* | TGTTGCATGAGAAAACGCCA | | | GCCTTGCAGCACTTCTCTAC |
| *HP* | TAGGGCGTGTGGGTTATGTT | | | AGATCCCAGTCGCATACCAG |
| *TF* | TCCCTTCTCATACCGTCGTG | | | CCACTTCACAGGCTTGCATT |
| *APOA1* | AAGCTCCTTGACAACTGGGA | | | CTCCTGCCACTTCTTCTGGA |
| *APOA2* | ACCGTGACTGACTATGGCAA | | | CAGGCTGTGTTCCAAGTTCC |
| *TLR5* | GAAAGGCTGCTGATGACACC | | | GGTGGAATAGGAGTAGGGGC |
| *FGA* | GAGGCCTAACAACCCAGACT | | | TTTAGAGCATGAACGACGCG |
| *GC* | GAAACACCAGCCACAGGAAT | | | GGTACAGCAGGACCCTACCA |
| **List of primers used for promoter construction** | | | | |
| Human Genes | Forward Primer Sequences (5’-3’) | | | Reverse Primer Sequences (5’-3’) |
| *ALB* | ATTTGGGACTTAACTCTTTC | | | CTTTGTATTTCATTGGCTG |
| *HP* | AAGAGCAATCAGAGAAGAC | | | CCAGTAACAAAACCACAAAC |
| *TF* | CACGTCCTCTCGGCGTCAA | | | AGGGTCCGGCCCAAGCTTT |
| **List of primers used for ChIP assays** | | | | |
| *ALB* | TGGAGAAAACAGTTCCAGATGGT | | | TGGCTGCCAACCGATTACAA |
| *HP* | AGATTGATGGTTCCTGCCGC | | | ATGACCCACAAACCTGCCAA |
| *TF* | TTGCCTTTTCCGGTGGAAGT | | | CTGCTCACCTCCACGTTTGA |
| **The sequences of shRNA oligonucleotides for gene knockdown.** | | | | |
| **shRNA oligonucleotides** | | **Sequences (5’-3’)** | | |
| Human scramble shRNA | | Top strand | GATCCGTTCTCCGAACGTGTCACGTAATTCAAGAGATTACGTGACACGTTCGGAGAATTTTTTC | |
|  |  | Bottom strand | AATTGAAAAAATTCTCCGAACGTGTCACGTAATCTCTTGAATTACGTGACACGTTCGGAGAACG | |
| Human Bmal1 shRNA | | Top strand | GATCCGCTTCTAGGCACATCGTGTTATTTCAAGAGAATAACACGATGTGCCTAGAAGTTTTTTG | |
|  |  | Bottom strand | AATTCAAAAAACTTCTAGGCACATCGTGTTATTCTCTTGAAATAACACGATGTGCCTAGAAGCG | |
| Mouse scramble shRNA | | Top strand | TGCTGAAATGTACTGCGCGTGGAGACGTTTTGGCCACTGACTGACGTCTCCACGCAGTACATTT | |
|  |  | Bottom strand | CCTGAAATGTACTGCGTGGAGACGTCAGTCAGTGGCCAAAACGTCTCCACGCGCAGTACATTTC | |
| Mouse Bmal1 shRNA | | Top strand | TGCTGAATGTTGGCTTGTAGTTTGCTGTTTTGGCCACTGACTGACAGCAAACTAAGCCAACATT | |
|  |  | Bottom strand | CCTGAATGTTGGCTTAGTTTGCTGTCAGTCAGTGGCCAAAACAGCAAACTACAAGCCAACATTC | |
