# Supplementary material for: The circadian clock gene Bmal1 facilitates cisplatin-induced renal injury and hepatization
Source: Cell Death Dis. 2020 Jun 10;11(6):446. doi: 10.1038/s41419-020-2655-1 (PMC7287064; doi:10.1038/s41419-020-2655-1)
Supplement: Supplementary file 2 — Supplementary Figure Legends [file 41419_2020_2655_MOESM2_ESM.docx]

**Supplementary Figure Legends**

**Supplementary Fig. 1 Cisplatin regulates renal clock gene expression both *in vivo* and *in vitro*. a-d** RT-qPCR analyses of *Clock*, *Per1*, *Cry1* and *Cry2* mRNA expression in the kidney from mice described in Fig. 2. *n* = 5, *^*^P* < 0.05 and *^**^P* < 0.01 *vs.* CTL group, ^#^*P* < 0.05 and ^##^*P* < 0.01 *vs.* ZT1 CTL group. **e, f** Quantitative analyses of Fig. 2C. *n* = 5, *^**^P* < 0.01 *vs.* CTL group, ^##^*P* < 0.01 *vs.* ZT1 CTL group. **g** Quantitative analysis of BMAL1 protein levels in Fig. 2F. *n* = 3, *^*^P* < 0.05 and *^**^P* < 0.01 *vs.* vehicle group. **h** Quantitative analysis of Fig. 2G. *n* = 3, *^**^P* < 0.01 *vs.* vehicle group. All values are presented as the mean ± SD.

**Supplementary Fig. 2 Overexpression of Bmal1 accelerates cisplatin-induced renal injury *in vitro*.** HK-2 cells were infected with adenovirus expressing either Bmal1 CDS domain or GFP for 24 h, followed by treatment of either 20 μM cisplatin or vehicle for another 24 h. **a** Flow cytometry assays. **b** TUNEL assays. Pink: TUNEL-positive cells; Blue: DAPI. **c, d** RT-qPCR and western blot analyses of renal expression levels of BMAL1, BAX/BCL-2, KIM-1, NGAL and cleaved-Caspase 3. *n* =3, *^*^P* < 0.05 and *^**^P* < 0.01 *vs.* Ad-GFP group, ^##^*P* < 0.01 *vs.* Ad-GFP + cisplatin group. All values are presented as the mean ± SD.

**Supplementary Fig. 3 Overexpression of Bmal1 accelerates cisplatin-induced renal injury** ***in vivo* and *in vitro*. a-c** Quantitative analyses of Fig. 3G. n = 5, *^**^P* < 0.01 *vs.* CTL group. **d** Statistical analyses of Fig. S2A. **e** Quantitative analyses of Fig. S2B. **f-j** Quantitative analyses of Fig. S2D. *n* =3, *^**^P* < 0.01 *vs.* Ad-GFP group, ^##^*P* < 0.01 *vs.* Ad-GFP + Cisplatin group. All values are presented as the mean ± SD.

**Supplementary Fig. 4 Overexpression of Per2 modestly affects cisplatin-induced renal injury.** HK-2 cells were infected with adenovirus expressing either Per2 CDS domain or GFP for 24 h, followed by treatment of either 20 μM cisplatin or vehicle for another 24 h. **a** TUNEL assays. Pink: TUNEL-positive cells; Blue: DAPI. **b** Quantitative analyses of panel **a**. NS, no significant, *n* = 3, *vs.* Ad-GFP + cisplatin group. **c-f** RT-qPCR analyses of renal expression levels of *PER2*, *BAX/BCL-2*, *KIM-1* and *NGAL*. *n* =3, *^**^P* < 0.01 *vs.* Ad-GFP group, ^##^*P* < 0.01 *vs.* Ad-GFP + cisplatin group. All values are presented as the mean ± SD.

**Supplementary Fig. 5 Knockdown of Bmal1 alleviates cisplatin-induced renal injury *in vitro*.** HK-2 cells were transfected with Bmal1 shRNA or scramble shRNA for 24 h, followed by treatment of either 20 μM cisplatin or vehicle for another 24 h. **a** Flow cytometry assays. **b** TUNEL assays. Pink: TUNEL-positive cells, Blue: DAPI. **c, d** RT-qPCR and western blot analyses of renal expression levels of BMAL1, BAX/BCL-2, KIM-1, NGAL and cleaved-Caspase 3,. *n* = 3, *^**^P* < 0.01 *vs.* scramble shRNA group, ^##^*P* < 0.01 *vs.* scramble shRNA + cisplatin group. All values are presented as the mean ± SD.

**Supplementary Fig. 6 Knockdown of Bmal1 alleviates cisplatin-induced renal injury *in vivo* and *in vitro*. a-c** Quantitative analyses of Fig. 4G. *n* = 5, *^*^P* < 0.05 and *^**^P* < 0.01 *vs.* CTL group. **d** Statistical analyses of Fig. S5A. **e** Quantitative analyses of Fig S5B. **f-j** Quantitative analyses of Fig. S5D. *n* =3, *^**^P* < 0.01 *vs.* scra shRNA group, ^##^*P* < 0.01 *vs.* scra shRNA + Cisplatin group. All values are presented as the mean ± SD.

**Supplementary Fig. 7 Bmal1 facilitates cisplatin-induced renal hepatization. a** Quantitative analysis of semi-quantitative RT-PCR performed in Fig. 5A. *n* = 6, *^**^P* < 0.01 *vs.* CTL group. HK-2 cells were treated as described in Fig. S2. **b** Western blot analysis of expression level of BMAL1 in HK-2 cells. **c** Quantitative analyses of Fig. 5C and S7B. *n* = 3, *^*^P* <0.05 and*^**^P* < 0.01 *vs.* Ad-GFP group. Mice were treated as described in Fig. 3. *n* = 5 for each group. **d** Western blot analysis of renal expression level of Bmal1. **e** Quantitative analyses of Fig. 5G and S7D. *n* = 5 *^**^P* < 0.01 *vs.* Ad-GFP + Cisplatin group. HK-2 cells were treated as described in Fig. S5. **f** Western blot analysis of protein expression level of BMAL1 in HK-2 cells. **g** Quantitative analyses of Fig. 6B and S7F. *n* = 3, *^**^P* < 0.01 *vs.* scra shRNA group, ^##^*P* < 0.01 *vs.* scra shRNA + Cisplatin group. Mice were treated as described in Fig. 4. *n* = 5 for each group. **h** Western blot analysis of renal expression level of Bmal1. **i** Quantitative analyses of Fig. 6F and S7H. *n* = 5 *^**^P* < 0.01 *vs.* Ad-scra shRNA + Cisplatin group. For panel **j** and **k**, HK-2 cells were treated as described in Fig. S4. **j** RT-qPCR analyses of mRNA expression levels of *PER2*, *ALB*, *HP* and *TF* in HK-2 cells. **k** ELISA analyses of the supernatant levels of ALB, HP and TF. *n* = 3, *^**^P* < 0.01 *vs.* Ad-GFP group, ^##^*P* < 0.01 *vs.* Ad-GFP + cisplatin group. All values are presented as the mean ± SD.
