# Supplementary figures and images for: The circadian clock gene Bmal1 facilitates cisplatin-induced renal injury and hepatization
Source: Cell Death Dis. 2020 Jun 10;11(6):446. doi: 10.1038/s41419-020-2655-1 (PMC7287064; doi:10.1038/s41419-020-2655-1)

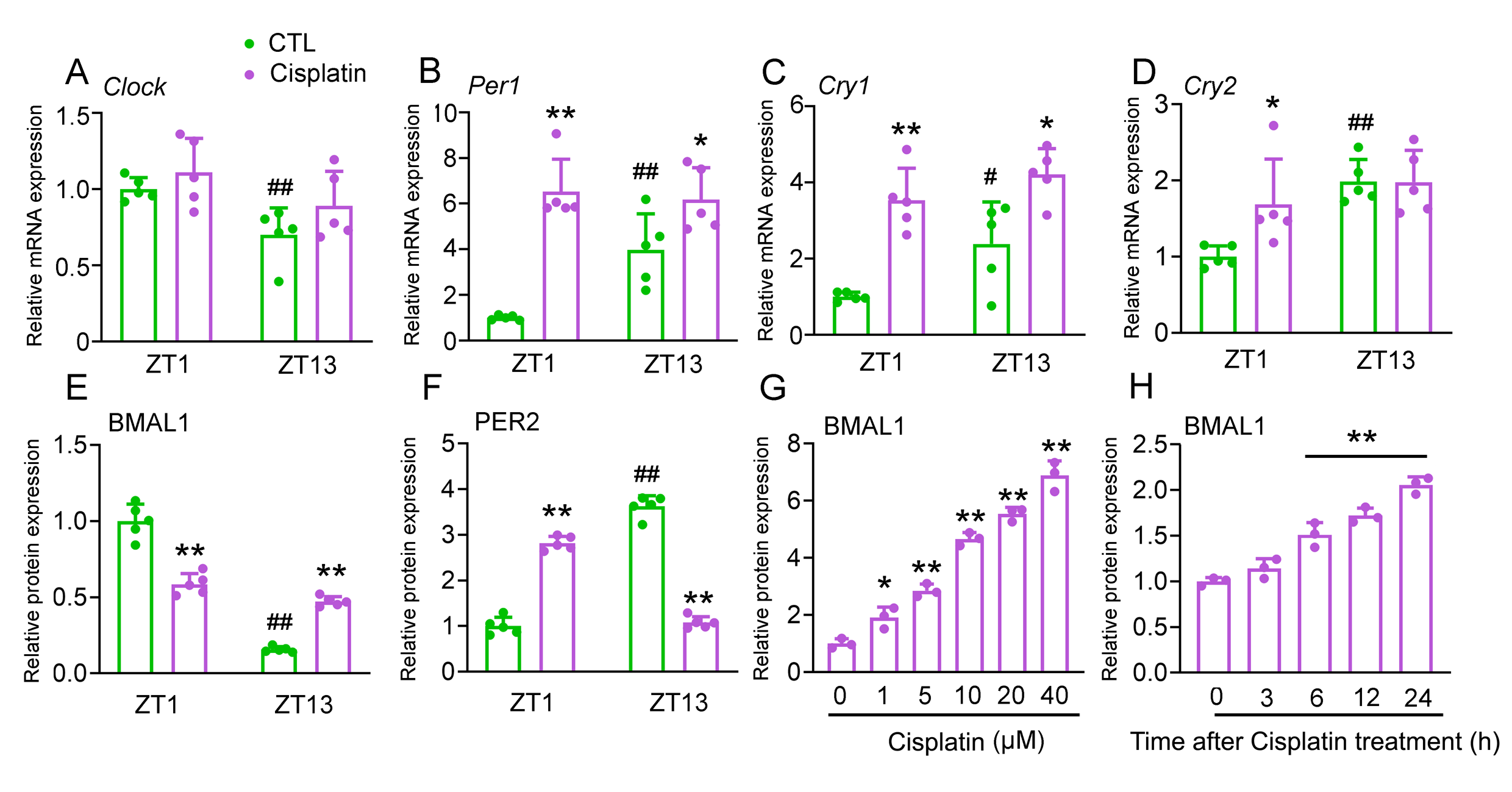

Supplement: Supplementary file 3 — Supplementary Figure 1 [file 41419_2020_2655_MOESM3_ESM.tif]

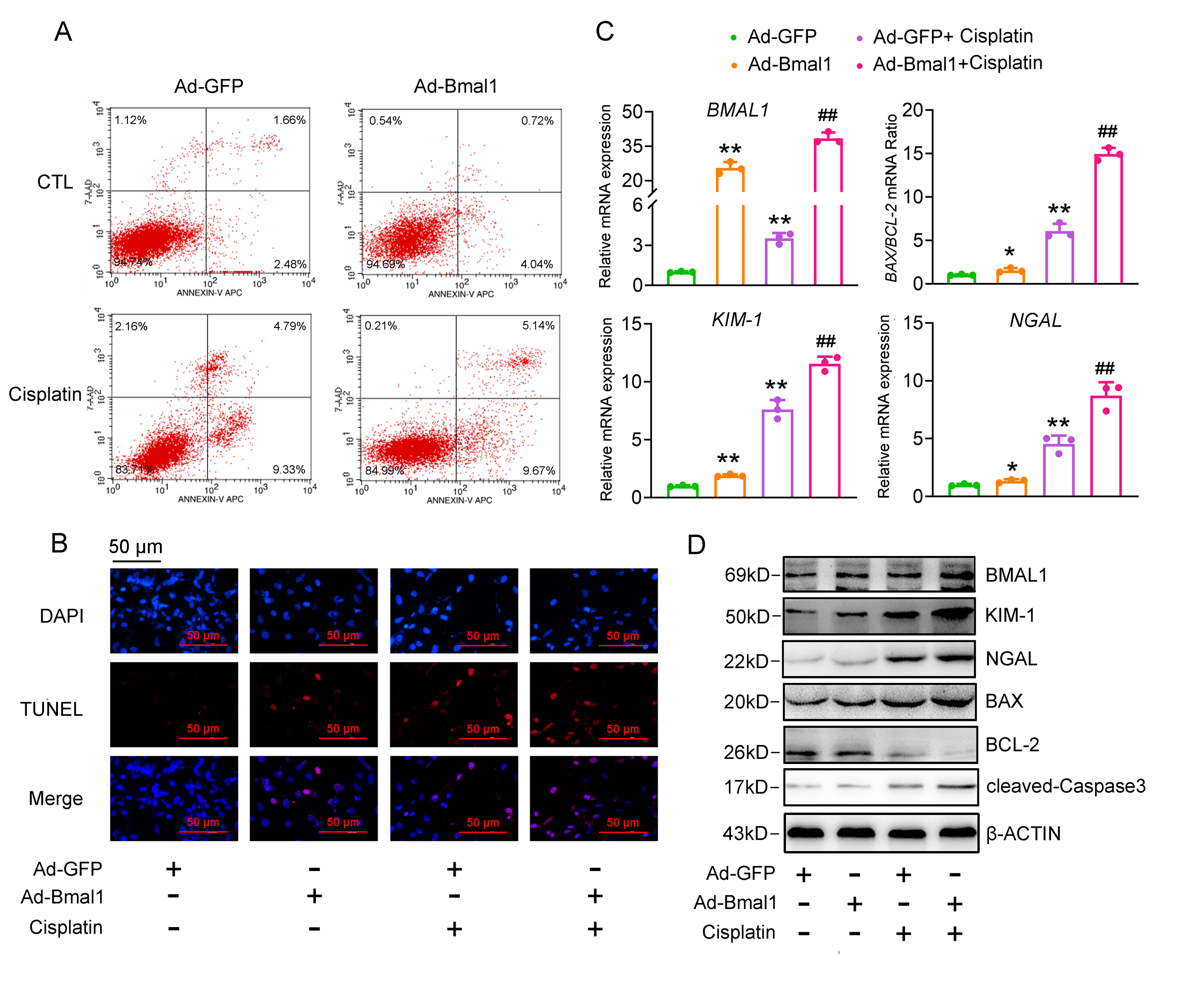

Supplement: Supplementary file 4 — Supplementary Figure 2 [file 41419_2020_2655_MOESM4_ESM.tif]

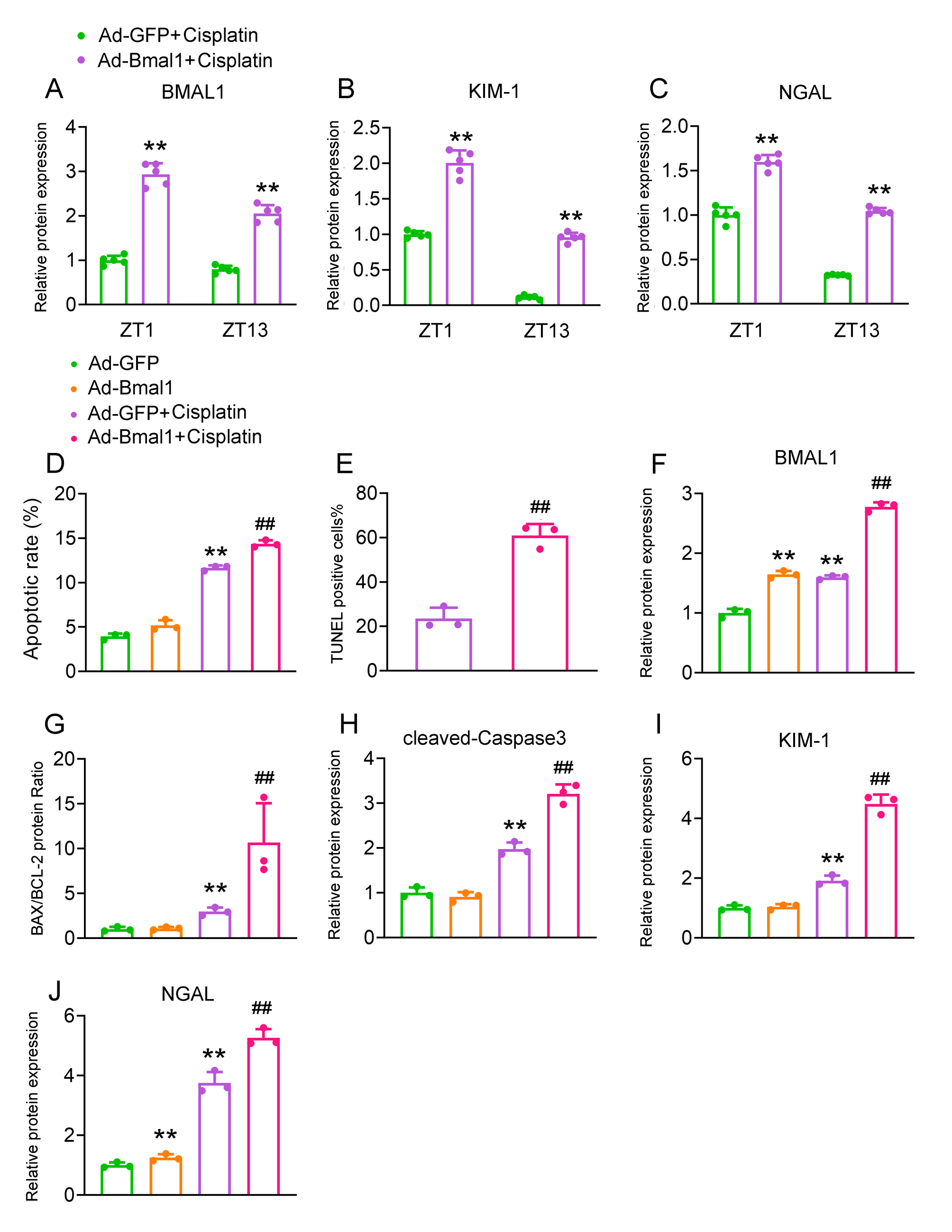

Supplement: Supplementary file 5 — Supplementary Figure 3 [file 41419_2020_2655_MOESM5_ESM.tif]

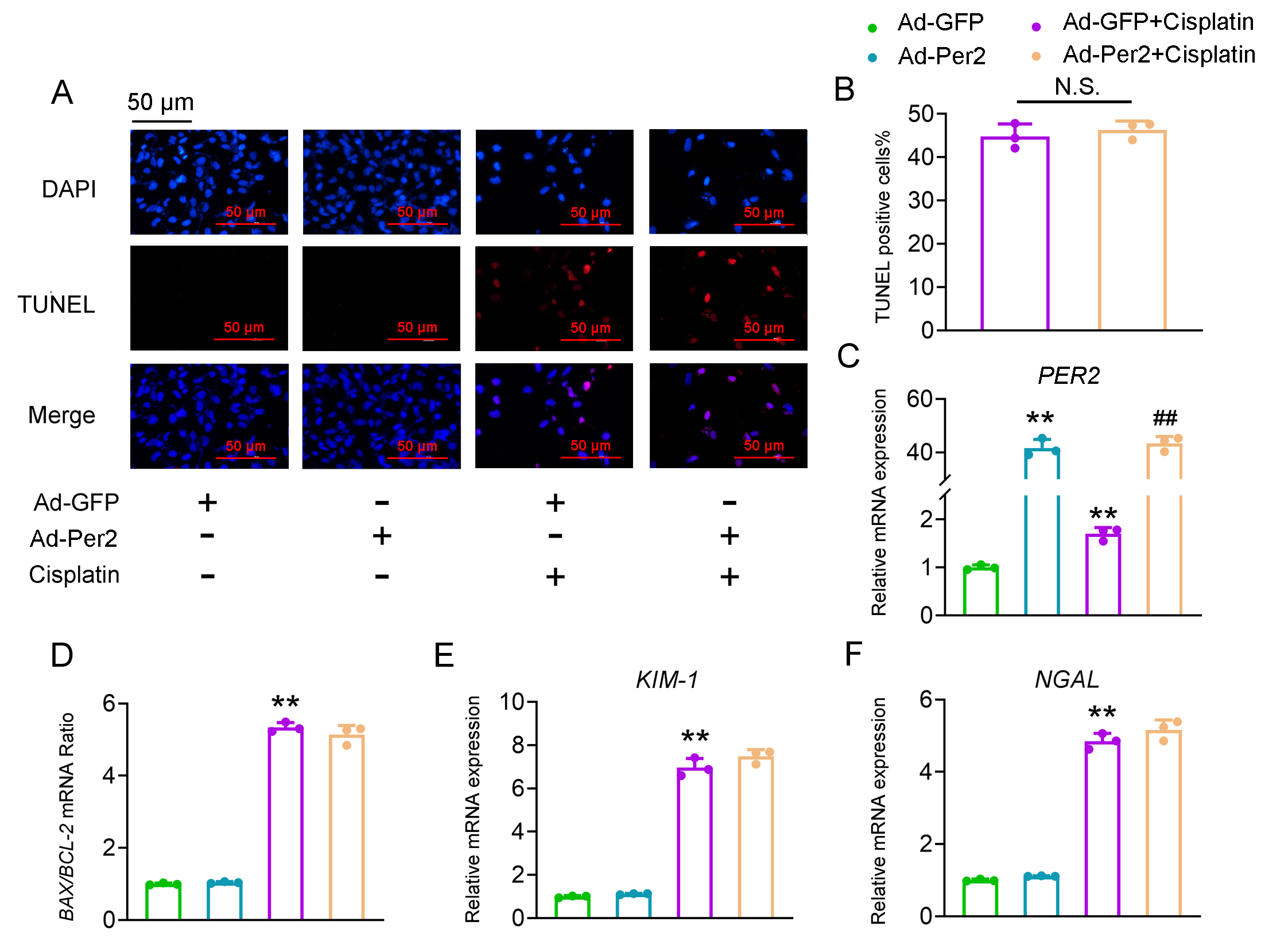

Supplement: Supplementary file 6 — Supplementary Figure 4 [file 41419_2020_2655_MOESM6_ESM.tif]

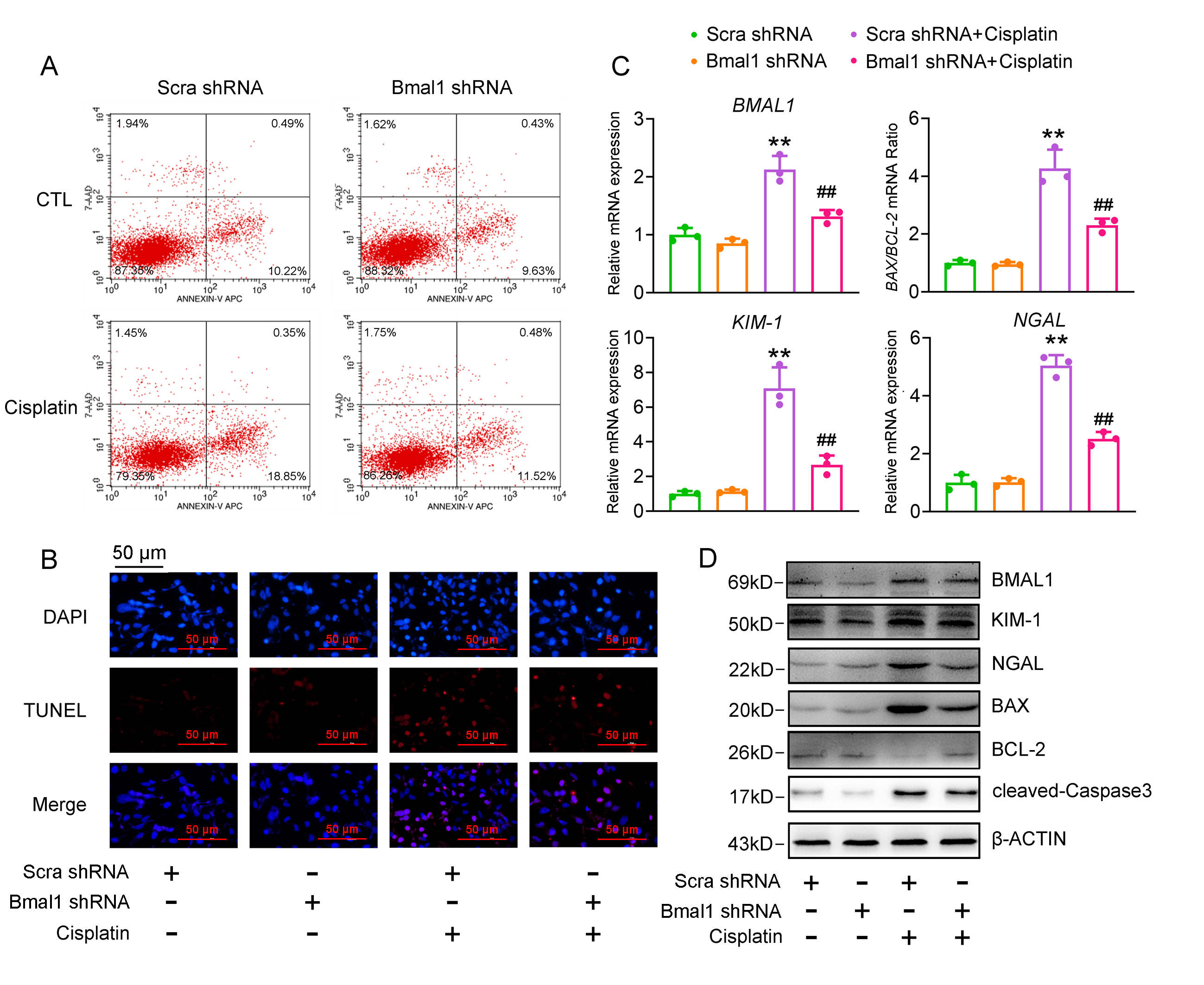

Supplement: Supplementary file 7 — Supplementary Figure 5 [file 41419_2020_2655_MOESM7_ESM.tif]

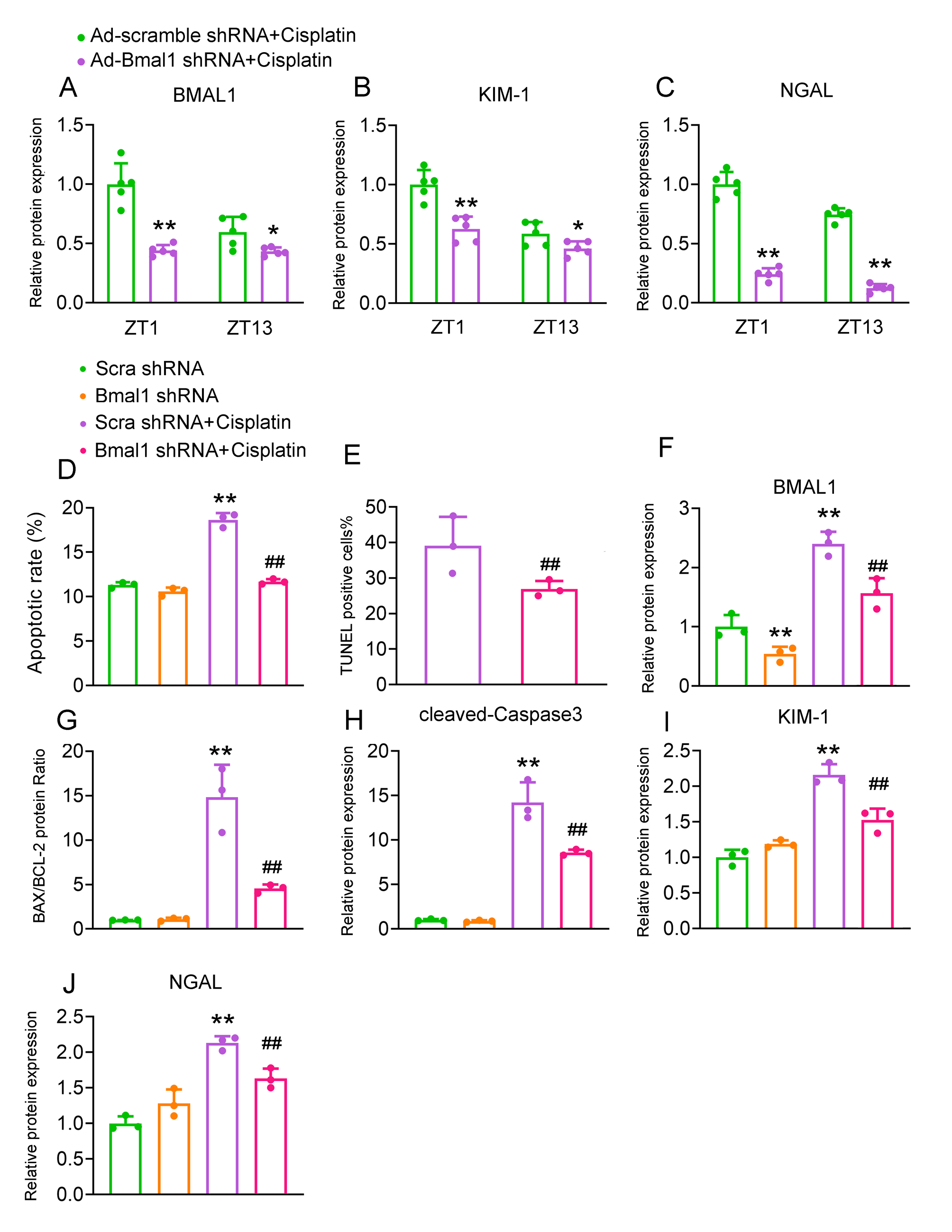

Supplement: Supplementary file 8 — Supplementary Figure 6 [file 41419_2020_2655_MOESM8_ESM.tif]

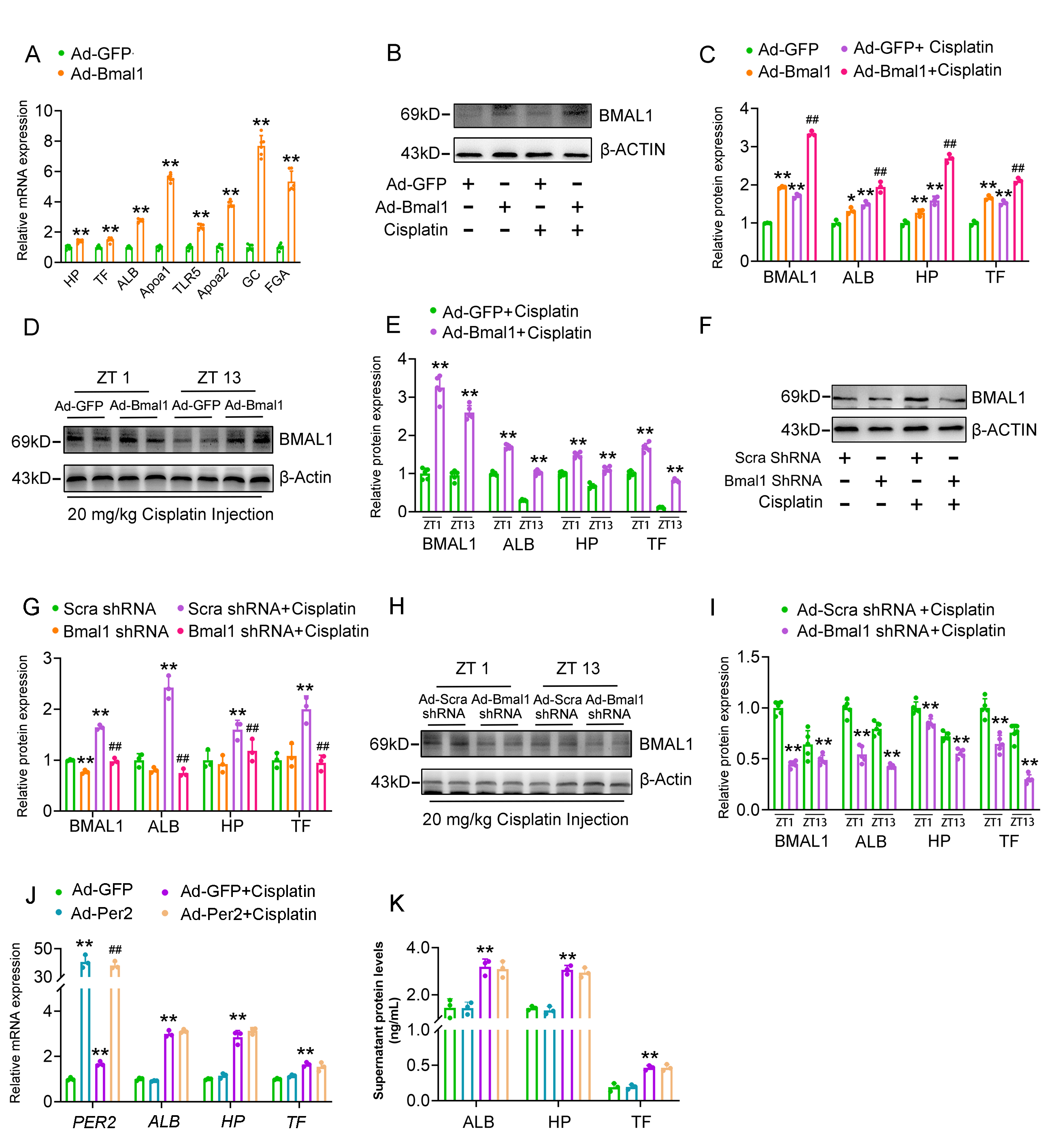

Supplement: Supplementary file 9 — Supplementary Figure 7 [file 41419_2020_2655_MOESM9_ESM.tif]
